# Myeloperoxidase is a critical mediator of anthracycline-induced cardiomyopathy

**DOI:** 10.1007/s00395-023-01006-0

**Published:** 2023-09-01

**Authors:** Felix Sebastian Nettersheim, Johannes David Schlüter, Wiebke Kreuzberg, Dennis Mehrkens, Simon Grimm, Harshal Nemade, Simon Braumann, Alexander Hof, Henning Guthoff, Vera Peters, Friedrich Felix Hoyer, Yulia Kargapolova, Jan-Wilm Lackmann, Stefan Müller, Christian P. Pallasch, Michael Hallek, Agapios Sachinidis, Matti Adam, Holger Winkels, Stephan Baldus, Simon Geißen, Martin Mollenhauer

**Affiliations:** 1 Department of Cardiology, Faculty of Medicine and University Hospital Cologne, University of Cologne, Kerpener Str. 62, 50937 Cologne, Germany; 2 Center for Molecular Medicine Cologne (CMMC), University of Cologne, Cologne, Germany; 3 CECAD, Faculty of Mathematics and Natural Sciences, University of Cologne, Cologne, Germany; 4 CECAD, Faculty of Medicine and University Hospital Cologne, University of Cologne, Cologne, Germany; 5 Department I of Internal Medicine, Center for Integrated Oncology (CIO) Köln-Bonn, Cologne, Germany; 6 Institute of Neurophysiology, Faculty of Medicine, University of Cologne, Cologne, Germany

**Keywords:** Doxorubicin, Cardiotoxicity, AICM, MPO, Polymorphonuclear neutrophils

## Abstract

Cardiotoxicity is a major complication of anthracycline therapy that negatively impacts prognosis. Effective pharmacotherapies for prevention of anthracycline-induced cardiomyopathy (AICM) are currently lacking. Increased plasma levels of the neutrophil-derived enzyme myeloperoxidase (MPO) predict occurrence of AICM in humans. We hypothesized that MPO release causally contributes to AICM. Mice intravenously injected with the anthracycline doxorubicin (DOX) exhibited higher neutrophil counts and MPO levels in the circulation and cardiac tissue compared to saline (NaCl)-treated controls. Neutrophil-like HL-60 cells exhibited increased MPO release upon exposition to DOX. DOX induced extensive nitrosative stress in cardiac tissue alongside with increased carbonylation of sarcomeric proteins in wildtype but not in *Mpo*^−/−^ mice. Accordingly, co-treatment of human induced pluripotent stem cell-derived cardiomyocytes (hiPSC-CMs) with DOX and MPO aggravated loss of hiPSC-CM-contractility compared to DOX treatment alone. DOX-treated animals exhibited pronounced cardiac apoptosis and inflammation, which was attenuated in MPO-deficient animals. Finally, genetic MPO deficiency and pharmacological MPO inhibition protected mice from the development of AICM. The anticancer efficacy of DOX was unaffected by MPO deficiency. Herein we identify MPO as a critical mediator of AICM. We demonstrate that DOX induces cardiac neutrophil infiltration and release of MPO, which directly impairs cardiac contractility through promoting oxidation of sarcomeric proteins, cardiac inflammation and cardiomyocyte apoptosis. MPO thus emerges as a promising pharmacological target for prevention of AICM.

**Supplementary Information:**

The online version contains supplementary material available at 10.1007/s00395-023-01006-0.

## Introduction

Anthracyclines are a class of antibiotics with high antitumour activity that were first isolated from *Streptomyces peucetius* in the early 1960s [15]. Anthracyclines have become a cornerstone of chemotherapy [34] and are included in the world health organization model list of essential medicines [58]. However, their clinical application is limited by a substantial risk of cardiotoxicity [53]. Anthracycline-induced cardiomyopathy (AICM)—the most prevalent form of chemotherapy-related heart disease—occurs dose-dependently and predominantly manifests as systolic heart failure [8, 42, 55]. Onset of AICM can be acute (immediately after infusion), early (within the first year), or late (several years after treatment) [60]. A recent study reports an incidence of 9% in patients exposed to anthracyclines and revealed that almost all (98%) cases occur within the first year with a median time to onset of 3.5 months [7]. AICM is associated with a high cardiovascular mortality that may affect long-term prognosis of anthracycline-treated cancer survivors [19]. Although several disease-mediating mechanisms have been identified in preclinical studies, pharmacological strategies to prevent AICM are missing [8, 42]. Currently, dexrazoxane is the only clinically approved compound for prevention of anthracycline-related cardiotoxicity [8, 42]. Dexrazoxane protects from AICM [32] by reducing mitochondrial oxygen radical formation [49] and inhibiting topoisomerase 2 [59]. Yet, due to concerns that dexrazoxane might reduce antitumour efficacy of anthracyclines and cause secondary malignancies, approval is restricted to selected patients [8, 42].

Increased plasma levels of the neutrophil-derived enzyme myeloperoxidase (MPO) were recently shown to predict cardiotoxicity in DOX-treated breast cancer patients [29, 45]. Accordingly, prolonged cardiac neutrophil infiltration significantly contributes to acute AICM in mice [48]. MPO amplifies the oxidative potential of hydrogen peroxide by enzymatically converting it to highly reactive oxygen species (ROS), such as hypochlorous acid (HOCl) [41]. The critical role of MPO in several cardiovascular diseases, such as atherosclerosis, myocardial infarction, arrhythmia, and pulmonary hypertension [24, 46] led to clinical development of oral MPO inhibitors [40]. It is yet unknown whether MPO is implicated in the pathogenesis of AICM and might thus represent a potential target for preventive pharmacotherapy.

We herein demonstrate that MPO critically contributes to acute DOX-induced cardiotoxicity by enhancing oxidation of sarcomeric proteins and promoting cardiac inflammation as well as apoptosis through p38 mitogen-activated protein kinase (MAPK) signaling. Importantly, genetic ablation as well as pharmacological inhibition of MPO protected DOX-treated mice from development of cardiac dysfunction, suggesting further evaluation of MPO as a therapeutic target for AICM prevention.

## Methods

### Animals

Eight- to twelve-week-old *Mpo*^−/−^ mice [4] on C57BL/6J background and wildtype littermates (WT) of both sexes were used in all experiments. All animal studies were approved by the local Animal Care and Use Committees (Ministry for Environment, Agriculture, Conservation and Consumer Protection of the State of North Rhine-Westphalia: State Agency for Nature, Environment and Consumer Protection (LANUV), NRW, Germany) and conformed to the guidelines from Directive 2010/63/EU of the European Parliament on the protection of animals used for scientific purposes.

### Experimental design

DOX is administered intravenously (i.v.) to patients. However, previous studies investigating AICM in mice largely utilized intraperitoneal (i.p.) DOX injections [44], which induced gut damage, endotoxin leakage, systemic inflammation [9, 12, 56] and were associated with high mortality [21]. Besides those adverse effects, we were not able to induce significant cardiotoxicity in C57BL/6J mice by repetitive i.p. injections of DOX (5 mg/kg bodyweight weekly for up to 7 weeks; data not shown). Repetitive i.v. injections via the tail vein have been reported to be clinically better tolerated [31]. However, we observed tail necrosis in the majority of mice injected via the tail vein in a pilot experiment (data not shown). Consequently, we established a model, in which mice were slowly injected with a single bolus DOX (20 mg/kg bodyweight; dissolved in 0.9% saline at a concentration of 3 mg/ml) or 0.9% saline (NaCl; 6.67 ml/kg bodyweight) via a jugular vein catheter (Fig. S1A). Mice were deeply anaesthetized by isoflurane inhalation (Isofluran-Piramal^®^, Piramal Critical Care, Voorschoten, The Netherlands; 5% vol/vol for induction and 2% vol/vol for maintenance of anaesthesia) and subcutaneous injection of buprenorphine (TEMGESIC^®^, Indivior Europe Limited, Dublin, Ireland; 0.1 mg per kg body weight). The adequacy of the anaesthesia was confirmed by pedal reflex testing. A small catheter was inserted into the left jugular vein and DOX was slowly infused with a Perfusor^®^ compact S (Braun Melsungen AG, Melsungen, Germany) over 30 min. We neither observed procedure- nor treatment-related mortality, whereas mice intraperitoneally injected with the same dose exhibited high mortality (Fig. S1B). Blood was drawn before DOX/NaCl injection from the Vena facialis of isoflurane-anaesthetized mice. Mice were examined by echocardiography at the indicated time points and tissues harvested 7 or 14 days after DOX/NaCl injection. A detailed description of echocardiography can be found in the Supplementary methods.

### MPO inhibitor treatment

WT mice were intraperitoneally injected with the irreversible MPO inhibitor 4-Aminobenzoic acid hydrazide (4-ABAH, Sigma-Aldrich, St. Louis, MO, USA; 20 mg/kg bodyweight) dissolved in 10% DMSO (14 mg/ml) or vehicle 2 days before DOX/NaCl injection and every other day thereafter until organ dissection at day 14 post treatment. Such administration scheme has been previously reported to sufficiently inhibit MPO [54].

### Tissue harvesting and subsequent analyses

Deeply anaesthetized mice (inhalation of isoflurane and injection of buprenorphine as described in “Experimental design” section) were sacrificed by cardiac exsanguination. Subsequent to perfusion with heparin (50 IU/ml) in 1 × PBS, hearts were dissected. Detailed descriptions of tissue preparation and subsequent histological and molecular biological analyses are provided in the Supplementary Methods.

### Cell culture experiments

Dimethyl sulfoxide (DMSO) differentiated HL-60 cells and human induced pluripotent stem cell-derived cardiomyocytes (hiPSC-CMs) were studied in vitro. Cell culture experiments are described in the Supplementary Methods.

### Statistical analysis and artwork

Data are presented as mean ± SD. Shapiro–Wilk tests suggested that the data was overall normally distributed and, accordingly, parametric tests were used for statistical analysis. Differences between groups were evaluated using one-way or two-way repeated measures analysis of variance (ANOVA) with post-hoc Tukey’s test. A mixed-effects analysis was used instead of a two-way repeated measures ANOVA for analyses of diastolic echocardiography parameters since values could not be obtained in some mice due to insufficient acoustic (apical four chamber view) windows. Log-rank (Mantel-Cox) test was used to determine significant differences in survival. A value of *P* < 0.05 was considered statistically significant. All statistical analyses were performed using GraphPad Prism 9 (GraphPad Software, San Diego, CA, USA). Microsoft PowerPoint (Microsoft, Redmond, WA, USA) was used to create the figures.

## Results

### DOX induces cardiac neutrophil infiltration and MPO release

**Fig. 1:**
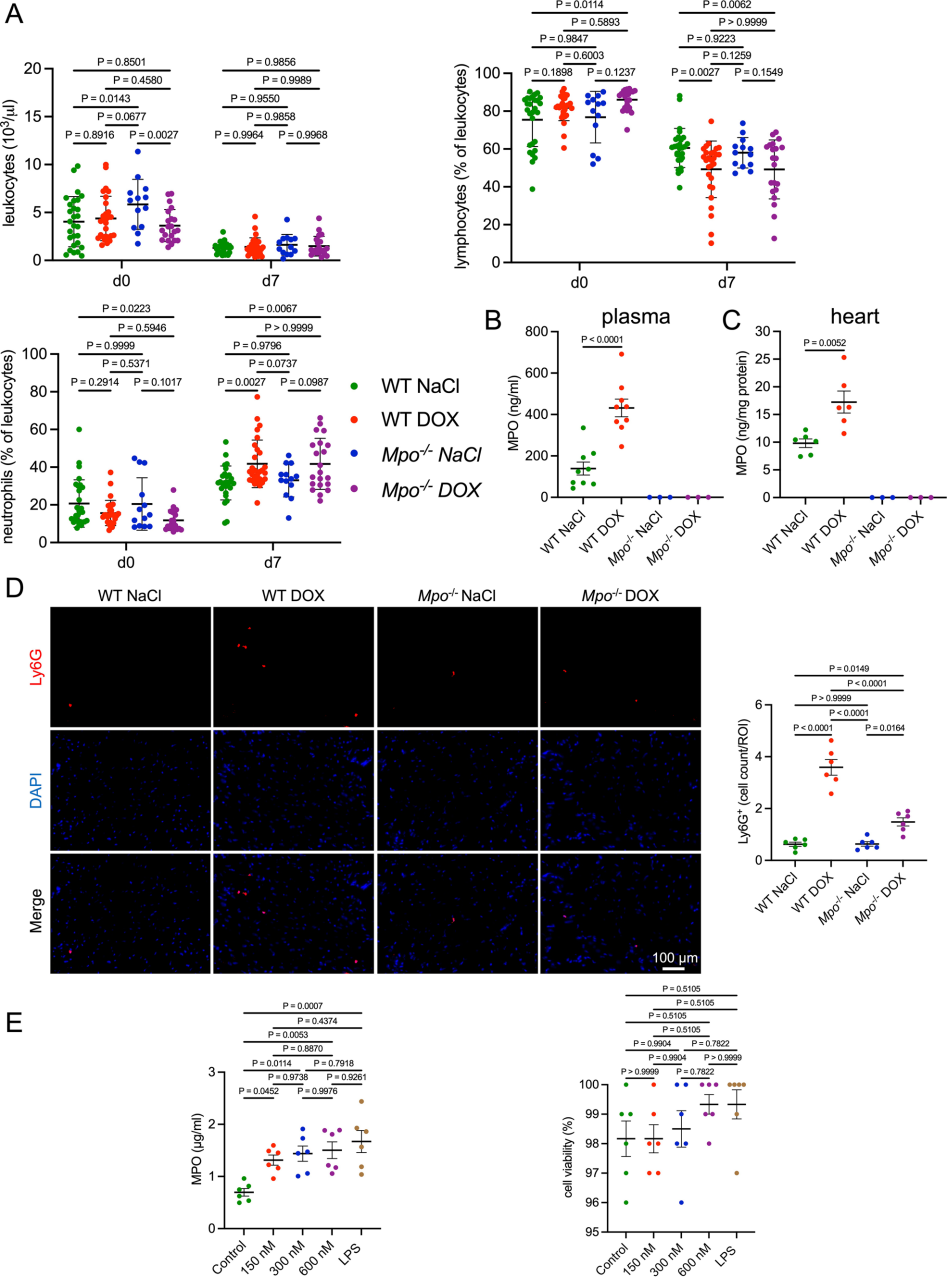
DOX increases systemic and cardiac MPO levels through induction of neutrophil recruitment and activation. **A** Blood leukocyte (10^3^/µl), lymphocyte (% of leukocytes), and neutrophil levels (% of leukocytes). d = day. n = 13–28 per group. **B** Blood MPO protein levels. n = 9 wildytpe (WT) mice and 3 *Mpo*^−/−^ negative controls per group. **C** Cardiac MPO protein levels. n = 6 wildytpe mice and 3 *Mpo*^−/−^ negative controls per group. **D** Representative Ly6G immunofluorescence stainings of cardiac sections and quantification of Ly6G^+^ cells (Ly6G^+^ cells per visual field). First row: Ly6G staining (red). Second row: DAPI-stained nuclei (blue). Third row: merged images. n = 6 per group. **E** MPO protein levels in the supernatant, and cell viability of neutrophil-like HL60-cells after 2-h treatment with DOX at different concentrations (150 nM, 300 nM, 600 nM), LPS (100 nM), or no treatment (Control). n = 6 per group. Data are expressed as mean ± SD. Statistical significance was determined by two-way repeated measures (**A**) or one-way (**B**–**E**) ANOVA with Tukey’s multiple comparisons test

To investigate the effects of DOX on systemic neutrophil levels and MPO release, we performed hematological analyses and measured plasma MPO levels. Before treatment (day 0), relevant differences in hematological parameters between WT and *MPO*^−/−^ mice were not detectable (Figs. 1a and S2). In line with previous reports [48], DOX-treated WT mice had lower circulating lymphocyte and higher neutrophil frequencies (% of leukocytes) compared to NaCl-treated controls 1 week after treatment, whereas total leukocyte counts did not differ (Fig. 1a). A similar trend was observed in *Mpo*^−/−^ mice. Additionally, DOX-treated WT mice showed a significant increase in basophil frequencies compared to DOX-treated *Mpo*^−/−^ mice and NaCl-treated mice (Fig. S2). Other leukocyte subsets, erythrocytes and platelets did not differ between groups. DOX was associated with a reduction in red blood cell distribution width (RDW). We observed consistent differences in most hematological parameters between the measurement at baseline and 7 days after treatment in all groups (lower numbers of leukocytes, lymphocytes, erythrocytes, platelets, lower hemoglobin, and higher numbers of neutrophils, monocytes, eosinophils, basophils at day seven compared to baseline; Figs. 1a and S2). We suggest that these differences were due to utilization of different blood collection methods (facial vein puncture at baseline vs. cardiac puncture at day 7), which has been shown to affect hematological parameters in C57BL/6J mice [20]. An additional analysis, in which values were normalized to the WT NaCl group at day 0 and day 7, respectively, confirmed that the observed differences in neutrophil and lymphocyte frequencies at day 7 were not affected by the batch effects between both measurement timepoints (Fig. S3). Whereas none of 22 tested cytokines were affected by i.v. DOX infusion (Fig. S4), plasma MPO levels were almost three-fold increased compared to NaCl treatment (Fig. 1b). Moreover, cardiac MPO levels were significantly higher in DOX versus NaCl-treated WT (Fig. 1c). In comparison to NaCl injection, DOX treatment was associated with an increase of cardiac Ly6G^+^ neutrophils in WT and to a significantly lesser extent in *Mpo*^−/−^ mice (Fig. 1d). Neutrophil-like HL60 cells exposed to DOX in vitro exhibited an increase in MPO release compared to untreated cells at all tested concentrations (150 nM, 300 nM, and 600 nM) which could not be explained by enhanced cell death rates (Fig. 1e). In conclusion, these data indicate that DOX induces MPO release by neutrophils and elicits a prolonged increase in systemic as well as in cardiac neutrophils, eventually leading to increased cardiac MPO levels.

### MPO deficiency attenuates cardiac dysfunction after exposure to DOX

**Fig. 2:**
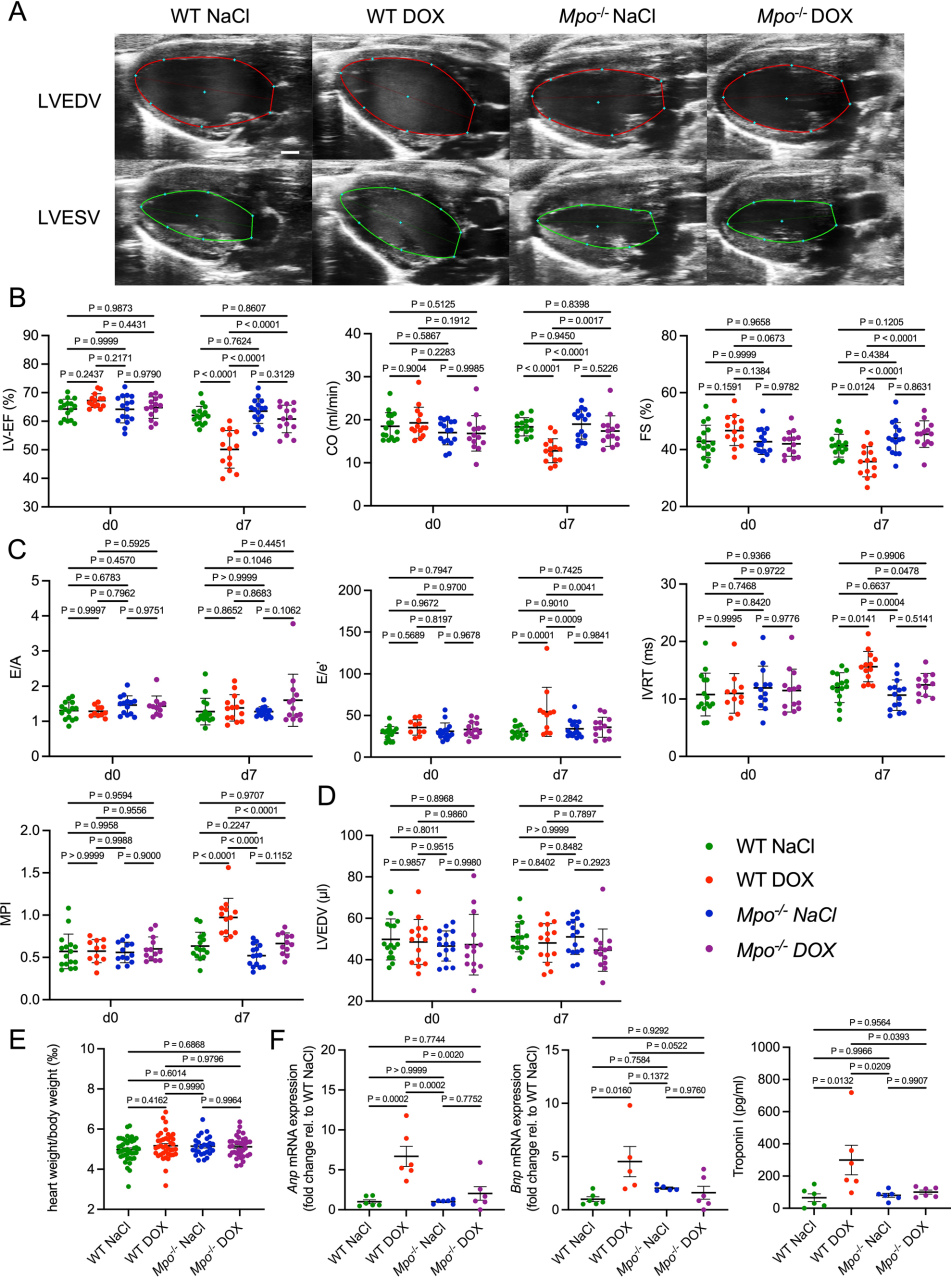
MPO deficiency mitigates development of acute AICM. **A** Representative 2D echocardiographic images of the left ventricle in the parasternal long axis view. LVEDV = left ventricular (LV) end-diastolic volume, LVESV = LV end-systolic volume. Scale bar indicates 1 mm. **B** Echocardiographic markers of systolic LV function: LV ejection fraction (LV-EF; %), cardiac output (CO; ml/min), fractional shortening (FS; %). d = day. n = 14–16 per group. **C** Markers of diastolic LV function: E/A, E/e’, isovolumetric relaxation time (IVRT, ms), myocardial performance index (MPI). n = 10–16 per group (some values were missing since measurements could not be obtained due to insufficient acoustic windows). **D** LVEDV (µl). n = 14–16 per group. **E** heart weight to body weight ratio (‰). n = 27–41 per group. **F** Cardiac *Anp* and *Bnp* mRNA expression (n = 5–6 per group) and blood Troponin I levels (n = 6 per group). **B**–**F** Data are expressed as mean ± SD. Statistical significance was determined by two-way repeated measures (**B**–**D**) or one-way (**E**, **F**) ANOVA with Tukey’s multiple comparisons test

To determine the functional relevance of MPO in AICM, we analyzed cardiac function by echocardiography 7 days post treatment. DOX-treated WT, but not *Mpo*^−/−^ mice, exhibited impairments of systolic (Fig. 2a, b) and diastolic (Fig. 2c) left ventricular (LV) function. Neither LV dilation (Fig. 2d) nor cardiac hypertrophy (Fig. 2e) could be observed after DOX-exposure, indicating an absence of significant cardiac remodeling. Yet, cardiac mRNA levels of atrial and B-type natriuretic peptide (*Anp* and *Bnp*), commonly used biomarkers for heart failure, and plasma levels of Troponin I, a marker of cardiomyocyte injury, were significantly increased in DOX- versus NaCl-treated WT, but not in *Mpo*^−/−^ mice (Fig. 2f). In summary, our results suggests that MPO is critically involved in mediating DOX-related cardiac dysfunction.

### Impact of MPO on DOX-related changes of cardiac protein expression

**Fig. 3:**
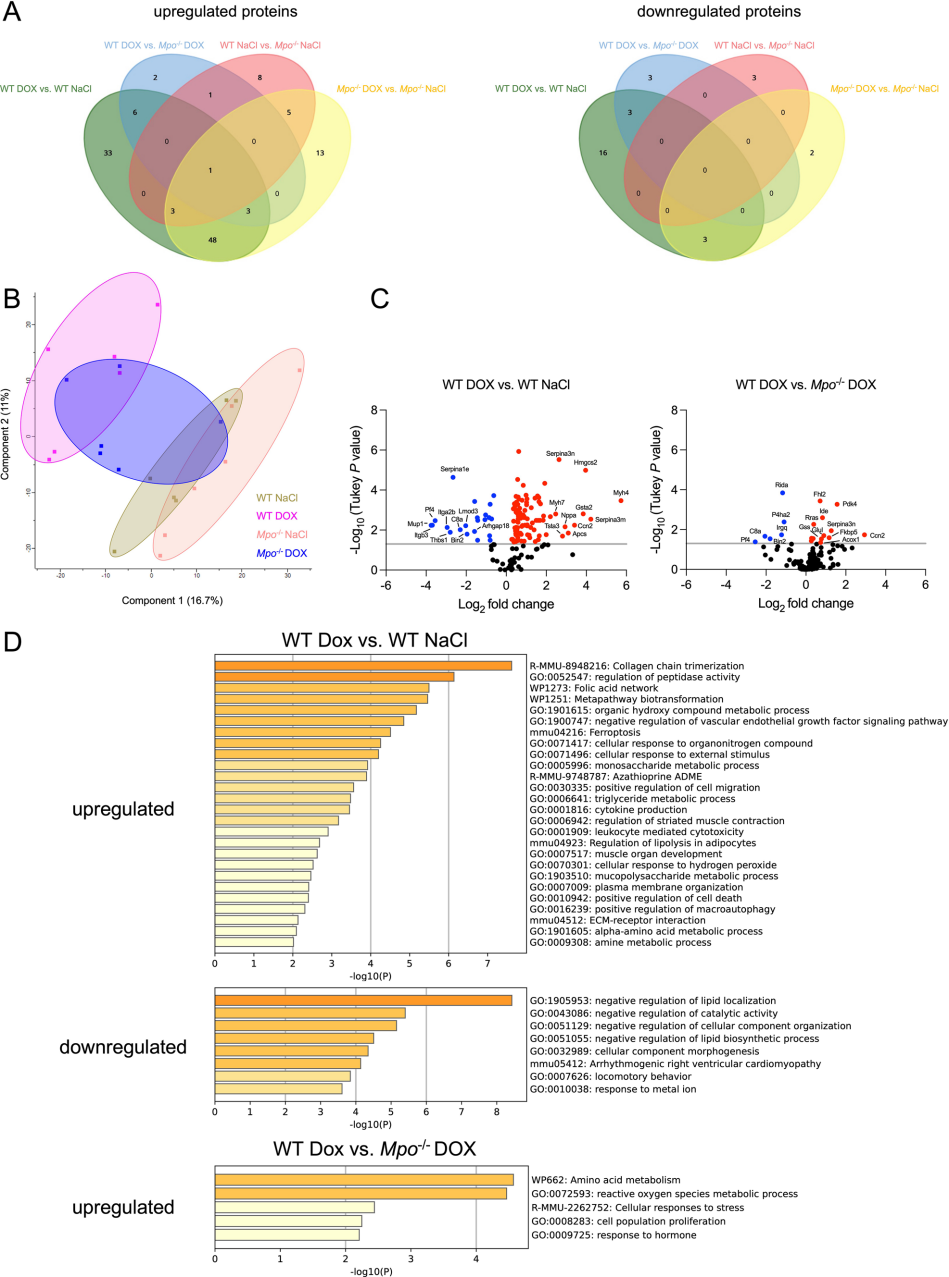
DOX-related changes in cardiac protein expression. **A** Venn diagrams of differentially expressed proteins. Up/downregulated proteins = proteins with significantly higher/lower expression in WT DOX versus WT NaCl (green), WT DOX versus *Mpo*^−/−^ DOX (blue), WT NaCl versus *Mpo*^−/−^ NaCl (red), and *Mpo*^−/−^ DOX versus *Mpo*^−/−^ NaCl (yellow). **B** Principal component analysis. DOX-treated WT clustered separately from NaCl-treated WT and *Mpo*^−/−^ mice, whereas the cluster of DOX-treated *Mpo*^−/−^ mice overlapped with both DOX-treated WT and NaCl-treated mice. Hence, only subtle changes in cardiac protein expression between DOX-treated WT and *Mpo*^−/−^ mice were detectable. **C** Volcano plots of differentially expressed proteins between DOX- versus NaCl-treated WT and DOX-treated WT versus *Mpo*^−/−^ mice. **D** Metascape pathway enrichment analysis (WT DOX vs. WT NaCl and WT DOX vs. *Mpo*^−/−^ DOX). Downregulated pathways in WT DOX versus *Mpo*^−/−^ DOX were not detectable. n = 6 per group. Statistical significance of differentially expressed proteins was determined by one-way ANOVA (FDR-adjusted) with Tukey’s multiple comparison test

We next performed mass spectrometry-based proteomics on cardiac tissue samples to determine DOX-related changes in protein expression. We first studied the impact of DOX treatment on cardiac protein expression in WT mice. 94 proteins were upregulated and 22 were downregulated in DOX- versus NaCl-treated WT hearts (Fig. 3a, Supplementary Excel Table). In MPO-deficient mice, DOX treatment caused less changes in protein abundance: DOX induced upregulation of 73 and downregulation of 5 proteins in *Mpo*^−/−^ mice. Abundances of 13 and 6 proteins were significantly higher and lower in DOX-treated WT compared to *Mpo*^−/−^ mice, respectively. Principal component analysis revealed distinct clusters of DOX- and NaCl-treated WT animals, whereas DOX-treated *Mpo*^−/−^ mice formed a cluster that overlapped with the WT DOX and both NaCl control groups (Fig. 3b). In line with previous reports, DOX induced expression of proteins related to atrophic cardiomyocytes (*e.g.* myosin heavy chain β isoforms MYH7 and MYH4) [57], which was not affected by MPO deficiency. Consistent with qPCR analysis (Fig. 2f), DOX induced an almost six-fold increase in cardiac ANP (NPPA) expression in WT that was not detectable in *Mpo*^−/−^ mice (Figs. 3c and S5A). Accordingly, expression of several proteins related to cardiac fibrosis and dysfunction, such as cellular communication network factor 2 (CCN2, also termed connective tissue growth factor = CTGF) [16, 52], pyruvate dehydrogenease kinase 4 (PDK4) [64], ras-related protein (RRAS)[33], and serpin peptidase inhibitor, clade A member 3 (SERPINA3)[14] was significantly higher in DOX-treated WT versus *Mpo*^−/−^ mice. Metascape pathway enrichment analysis revealed an upregulation of pathways related to fibrosis, leukocyte mediated cytotoxicity, cell death, cytokine production, response to organonitrogens, and autophagy in DOX- versus NaCl-treated WT but not in *Mpo*^−/−^ mice (Figs. 3d and S5B). Although the changes in cardiac protein expression between DOX-treated WT and *Mpo*^−/−^ mice were subtle, we were able to identify pathways, which were significantly less influenced by DOX treatment in MPO-deficient animals. Particularly, direct comparison between DOX-treated WT and *Mpo*^−/−^ mice showed enrichment in oxidative stress-related pathways in WT, but not in *Mpo*^−/−^ mice (Fig. 3d). Only few proteins and pathways were differentially expressed in NaCl-treated WT versus *Mpo*^−/−^ mice, which argues against a relevant impact of MPO deficiency on basal cardiac protein expression (Fig. S5A, B).

### MPO mediates DOX-related oxidation of sarcomeric proteins and reduces cardiomyocyte contractility

**Fig. 4:**
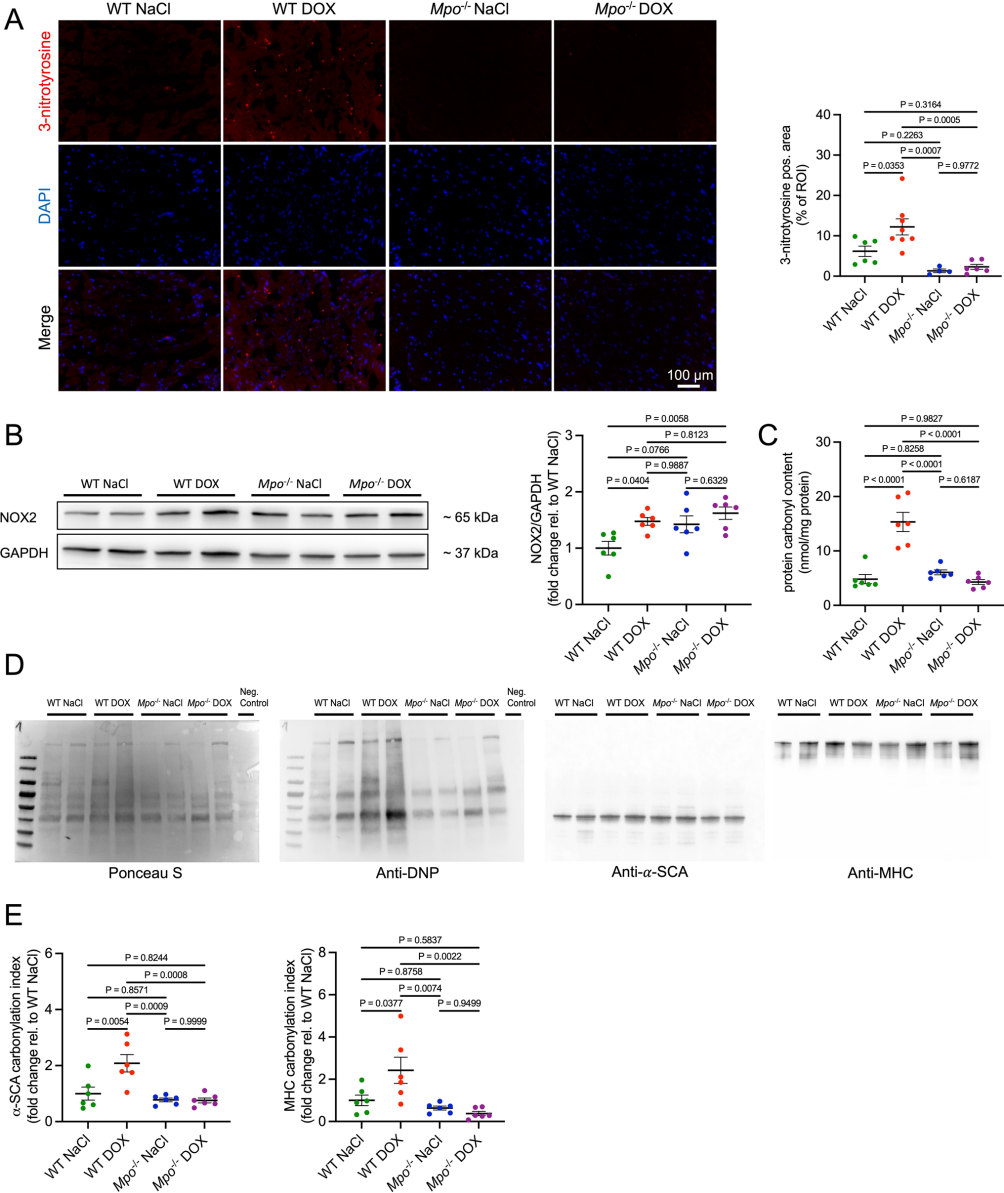
MPO induces oxidation of sarcomeric proteins after exposure to DOX. **A** Representative 3-nitrotyrosine immunofluorescence stainings of cardiac sections and quantification of 3-nitrotyrosine positive area (% of visual field). First row: 3-nitrotyrosine staining (red). Second row: DAPI-stained nuclei (blue). Third row: merged images. n = 4–8 per group. **B** Representative immunoblots of NOX2 in cardiac tissue samples and quantification of cardiac NOX2 protein expression. **C** Cardiac protein carbonylation (mmol/mg protein) as revealed by protein carbonyl assay. **D** Representative protein carbonyl immunoblots of cardiac tissue. **E** Carbonylation index of protein bands corresponding to α-sarcomeric actin (α-SCA) and myosin heavy chain (MHC). To determine the carbonylation indices, densities of anti-DNP-stained bands (carbonylated protein) were divided by densities of corresponding bands in the Ponceau S staining (total protein). **B**–**E** n = 6 per group. **A**–**E** Data are expressed as mean ± SD. Statistical significance was determined by one-way ANOVA with Tukey’s multiple comparisons test

Proteomic analyses suggested that MPO deficiency decreased the oxidative stress response in cardiac tissue after exposure to DOX. To confirm these findings, we histologically quantified cardiac levels of 3-nitrotyrosine, a marker of oxidative enzymatic MPO activity [41]. Whereas DOX induced cardiac 3-nitrotyrosine generation in WT hearts, such effect was not observed in *Mpo*^−/−^ mice (Fig. 4a). Cardiac expression of NADPH oxidase 2 (NOX2) was equally increased in both WT and *Mpo*^−/−^ mice compared to NaCl-treated controls (Fig. 4b). Cardiac mRNA expression of *Nox1*, *Nox3*, xanthine oxidase (*Xo*), Nitric oxide synthase 1 (*Nos1*) and *Nos*2 (Fig. S6A, C) as well as XO activity (Fig. S6B) did not differ between the groups, whereas *Nos3* mRNA levels were elevated in DOX-treated *Mpo*^−/−^ versus WT mice (Fig. S6C). Oxidative modifications of sarcomeric proteins contribute to contractile dysfunction in heart failure patients [6] and MPO has been shown to induce sarcomeric protein carbonylation in vitro [23]. In line with these data, we observed a DOX-related increase in cardiac protein carbonylation in WT but not in *Mpo*^−/−^ mice (Fig. 4c). Protein carbonyl immunoblot assays, in which carbonyl groups present in all proteins are derivatized to 2,4-dinitrophenylhydrazone (DNP-hydrazone) and subsequently stained with anti-DNP antibodies, indicated pronounced oxidation of protein bands corresponding to α-sarcomeric actin (α-SCA) and myosin heavy chain (MHC; Fig. 4d, e).

**Fig. 5:**
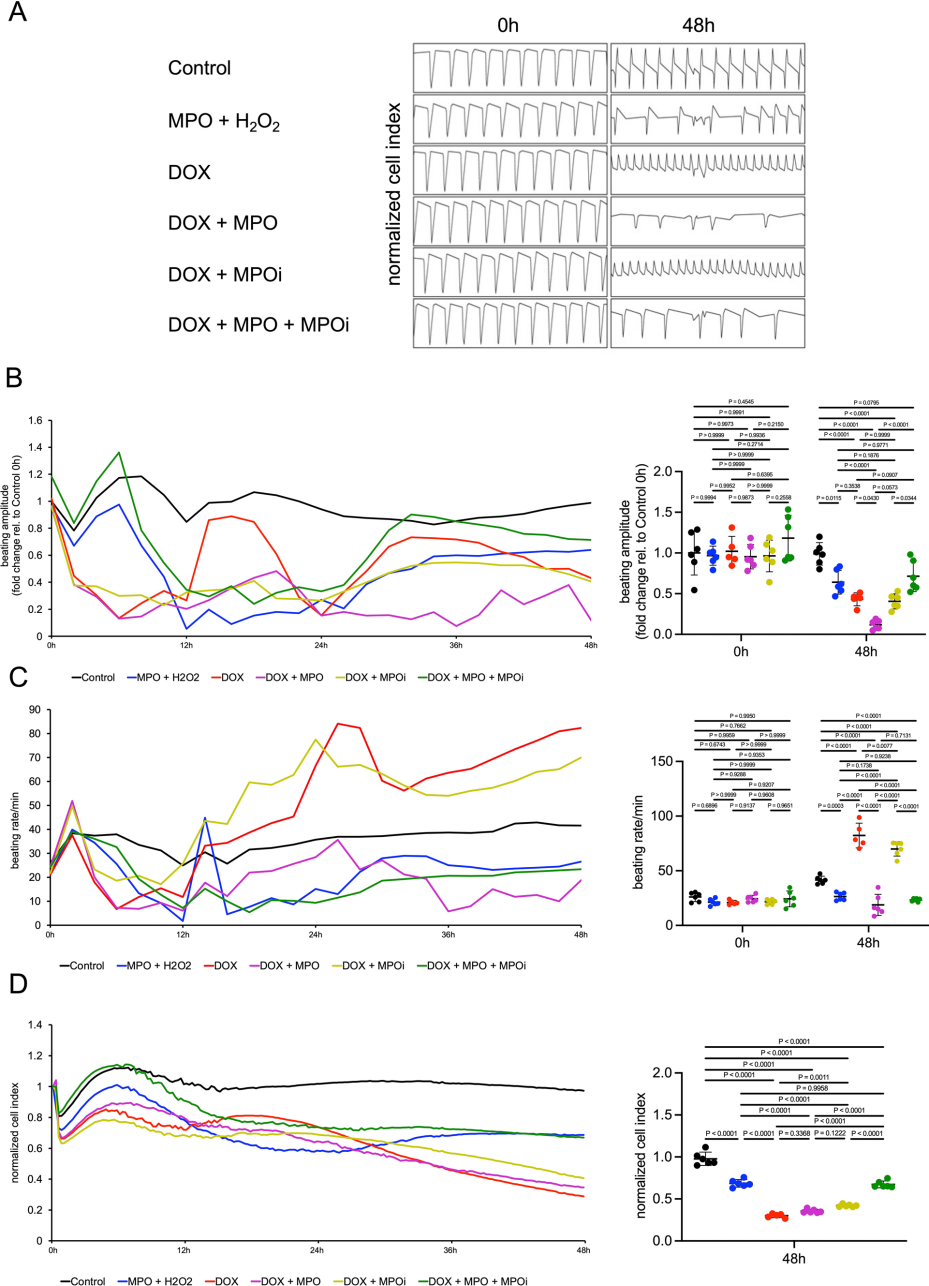
MPO amplifies DOX-related impairment of cardiomyocyte contractility. **A** Representative Real-Time Cell Analyzer recordings of induced pluripotent stem cell-derived cardiomyocytes. Cells were left untreated (Control) or exposed to MPO (10 µg/ml) + H_2_O_2_ (40 µM), DOX (156 nM), DOX + MPO, DOX + MPO inhibitor 4-Aminobenzoic acid hydrazide (MPOi, 50 µM), or DOX + MPO + MPOi for 48 h (h). **B** Beating amplitude initially decreased in all treatment conditions compared to Control. In contrast to all other groups, beating amplitude of cells exposed to DOX + MPO failed to recover after 24 h and was eventually significantly reduced. **C** Beating rates of cells treated with DOX with/without MPOi were significantly higher compared to Control, whereas treatment with MPO and H_2_O_2_ or MPO and DOX reduced the beating rate. **D** Normalized cell index (fraction of viable cells in relation to baseline values which were set to 1.0 for all wells). n = 5–6 per group. Data are expressed as mean ± SD. Statistical significance was determined by two-way repeated measures (**B**, **C**) and one-way (**D**) ANOVA with Tukey’s multiple comparisons test

To determine whether MPO-dependent protein oxidation after DOX treatment indeed translated to cardiomyocyte dysfunction, we exposed hiPSC-CMs to MPO + H_2_O_2_ (the substrate of MPO), DOX, DOX + MPO, DOX + MPO inhibitor 4-Aminobenzoic acid hydrazide (MPOi), or DOX + MPO + MPOi and recorded their beating profiles using the xCELLigence RTCA Cardio system. All treatments led to an initial reduction in beating amplitude compared to untreated cells (black), which was followed by a recovery from 24 h onwards. Cells exposed to DOX + MPO (purple) failed to recover and eventually showed a significant reduction in beating amplitude compared to cells exposed to DOX only or MPO and H_2_O_2_. This reduction was prevented by MPOi treatment (green; Fig. 5a, b). Cells treated with DOX with/without MPOi (red/dark yellow) exhibited significantly increased beating rates compared to control, whereas treatment with MPO and H_2_O_2_ (blue), or MPO and DOX (with/without MPOi) reduced the beating rate (Fig. 5a, c). The decrease in beating amplitude in DOX + MPO versus DOX treated cells (red vs. pink) was not explained by differences in cell viability, although additional MPOi treatment improved cell survival (Fig. 5d). In summary, these data confirm that MPO amplifies DOX-related contractile dysfunction of cardiomyocytes.

### Cardiomyocyte apoptosis and cardiac inflammation after DOX-treatment is mediated by MPO

**Fig. 6:**
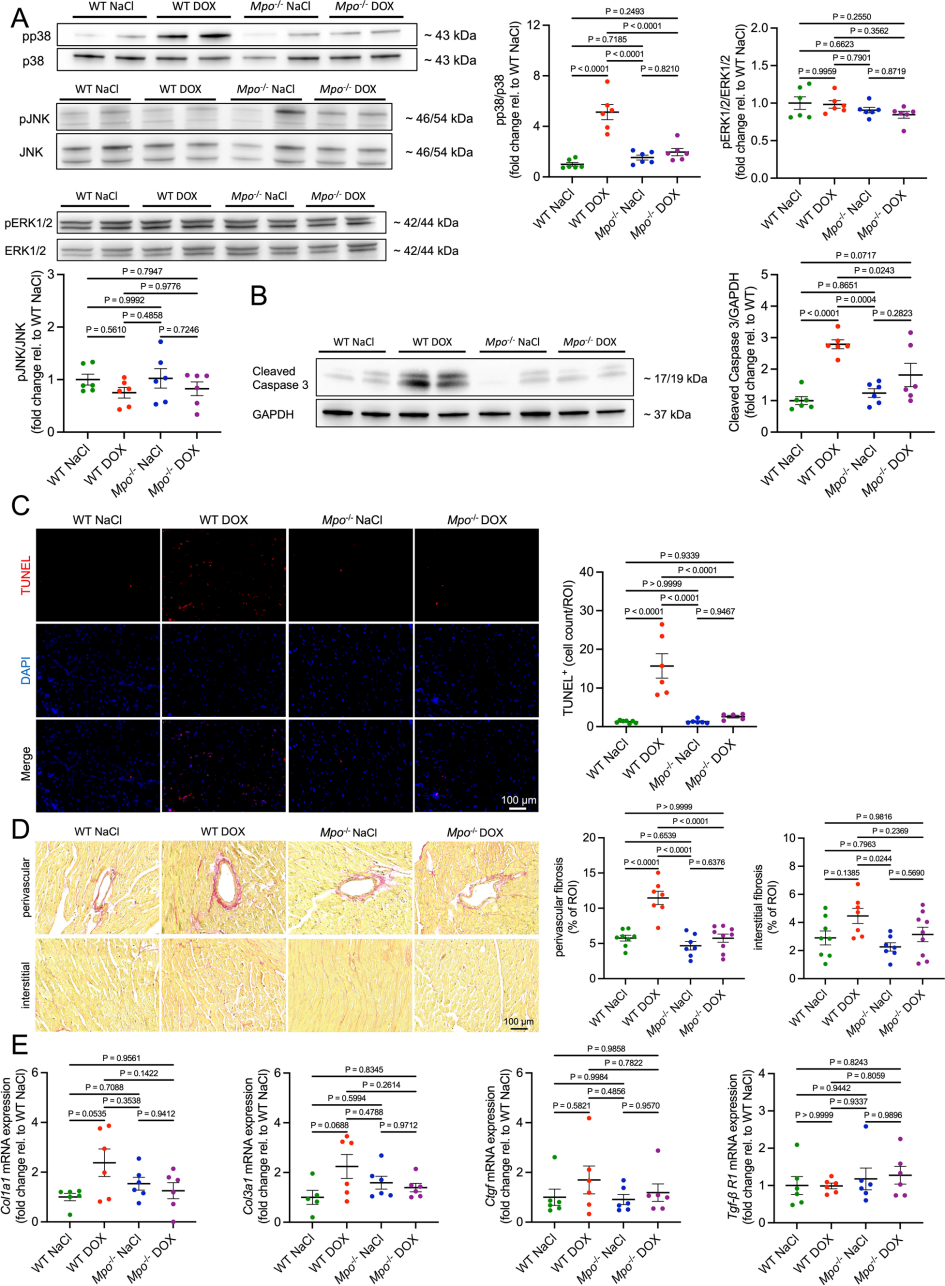
MPO mediates DOX-related induction of p38 signaling and cardiomyocyte apoptosis. Representative immunoblots and corresponding quantifications of phospho(p)/total p38, JNK, and ERK1/2 (**A**), and Cleaved Caspase 3 (**B**) in cardiac tissue. n = 6 per group. **C** Representative TUNEL stainings of cardiac sections and quantification of TUNEL^+^ cells per visual field. First row: TUNEL staining (red). Second row: DAPI-stained nuclei (blue). Third row: merged images. n = 5–7 per group. **D** Representative Picrosirius red stainings of cardiac sections and quantification of perivascular and interstitial fibrosis (red staining % of visual field). n = 7–9 per group. **E** Cardiac mRNA expression of fibrosis related genes: Collagen type 1 alpha 1 and 3 chains (*Col1a1*, *Col3a1*), connective tissue growth factor (*Ctgf*), and transforming growth factor β receptor 1 (*Tgf-β R1*). n = 5–6 per group. Data are expressed as mean ± SD. Statistical significance was determined by one-way ANOVA with Tukey’s multiple comparisons test

Proteomics indicated an upregulation in oxidative stress-, cell death-, fibrosis-, and autophagy-related pathways in cardiac tissue of DOX-treated WT versus *Mpo*^−/−^ mice and NaCl-treated controls. Considering that mitogen-activated protein kinases (MAPKs) and Signal Transducer and Activator of Transcription (STAT) proteins are known to be regulated by ROS and to affect cell death [5, 13, 51], we measured their phosphorylation in cardiac tissue by immunoblotting. Whereas cardiac phosphorylation of p38 was significantly increased in DOX-treated WT compared to all other groups, no differences in JNK-, ERK1/2-, STAT1-, and STAT3-phosphorylation were detected (Figs. 6a and S7A). In line with proteomics, Cleaved Caspase 3 immunoblot and Terminal deoxynucleotidyl transferase dUTP nick end labeling (TUNEL) staining indicated DOX-related induction of cardiac apoptosis, which was attenuated in *Mpo*^−/−^ mice (Fig. 6b, c). Next, we histologically quantified cardiac fibrosis to validate DOX-related enrichment in fibrosis pathways observed in proteomic analyses. Whereas perivascular fibrosis was significantly increased in DOX-treated WT compared to NaCl-treated controls and DOX-treated *Mpo*^−/−^ mice, only subtle differences in interstitial fibrosis could be detected (Fig. 6d). Accordingly, the WT DOX group exhibited only trendwise increases in mRNA expression of fibrosis-related genes (Fig. 6e). Together these data show an early (perivascular) fibrotic reaction in hearts of DOX-treated WT, that did unlikely contribute to the observed phenotype. Immunoblot analysis of the autophagy marker LC3A/B did not confirm DOX-related alterations in autophagy, as suggested by proteomics (Fig. S7B).

**Fig. 7:**
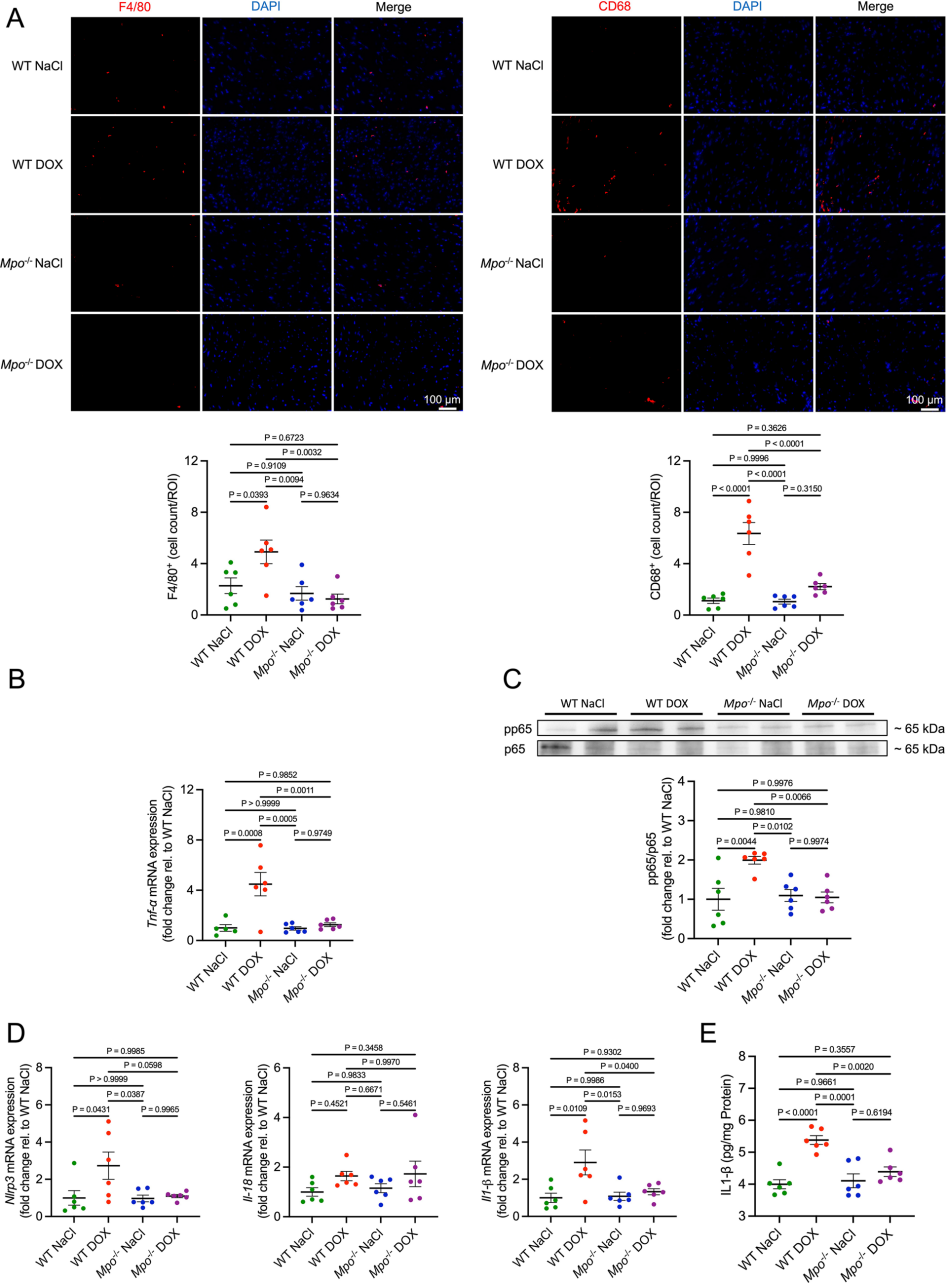
MPO triggers cardiac inflammation after DOX treatment. **A** Representative F4/80 and CD68 immunofluorescence stainings of cardiac sections and quantification of F4/80^+^ and CD68^+^ cells (F4/80^+^/CD68^+^ cells per visual field). First column: Ly6G/F4/80 staining (red). Second column: DAPI-stained nuclei (blue). Third column: merged images. n = 6 per group. **B** Tumor necrosis factor *α* (*Tnf-α*) mRNA levels in cardiac tissue. n = 5–6 per group. **C** Representative immunoblot and corresponding quantification of phospho(p)/total p65 in cardiac tissue. **D** NLR family pyrin domain containing 3 (*Nlrp3*), Interleukin 18 (*Il-18*) and 1-β (*Il1-β*) mRNA levels in cardiac tissue. **E** *IL1-β* protein levels in cardiac tissue. **C**–**E** n = 6 per group. Data are expressed as mean ± SD. Statistical significance was determined by one-way ANOVA with Tukey’s multiple comparisons test

We and others have shown that MPO mediates cardiac monocyte/macrophage recruitment and activation after myocardial infarction [2, 37]. Accordingly, DOX-treated WT exhibited an increase in cardiac F4/80^+^ and CD68^+^ macrophage counts compared to controls, that was not detectable in *Mpo*^−/−^ mice (Fig. 7a). Cardiac mRNA expression of the pro-inflammatory cytokine tumor necrosis factor alpha (*Tnf-α;* Fig. 7b), phosphorylation of the nuclear factor kappa B (NF-κB) subunit p65 (a marker of NF-κB activation, Fig. 7c), and cardiac mRNA expression of NLR Family Pyrin Domain Containing 3 (*Nlrp3*) and interleukin-1β (*Il1-β*; Fig. 7d) were increased in DOX-treated WT compared to all other groups. DOX-related overexpression of IL-1β was confirmed on protein level (Fig. 7e), whereas TNF-α was not detectable by ELISA. Cardiac mRNA expression of *Il-18*, *Il-6*, and *Il-10* did not differ between the groups (Figs. 7d and S8A). Cardiac mRNA expression of the leukocyte adhesion molecule intercellular adhesion molecule 1 (*Icam-1*) was significantly increased in the WT DOX group compared to all other groups (Fig. S8B). Additionally, DOX-treated WT exhibited marginal increases in cardiac mRNA expression of vascular cell adhesion molecule 1 (*Vcam-1*) and *E selectin* that did not reach statistical significance. DOX induced an increase in cardiac mRNA expression of chemokine (C-X-C motif) ligand 1 (*Cxcl*1), a mediator of neutrophil-recruitment, whereas expression of several other tested chemokines and chemokine receptors was not affected by DOX (Fig. S8C). In conclusion, our data indicates that MPO mediates DOX-related induction of p38-MAPK-signaling, apoptosis, and inflammation in cardiac tissue.

### Pharmacological MPO inhibition protects from AICM

**Fig. 8:**
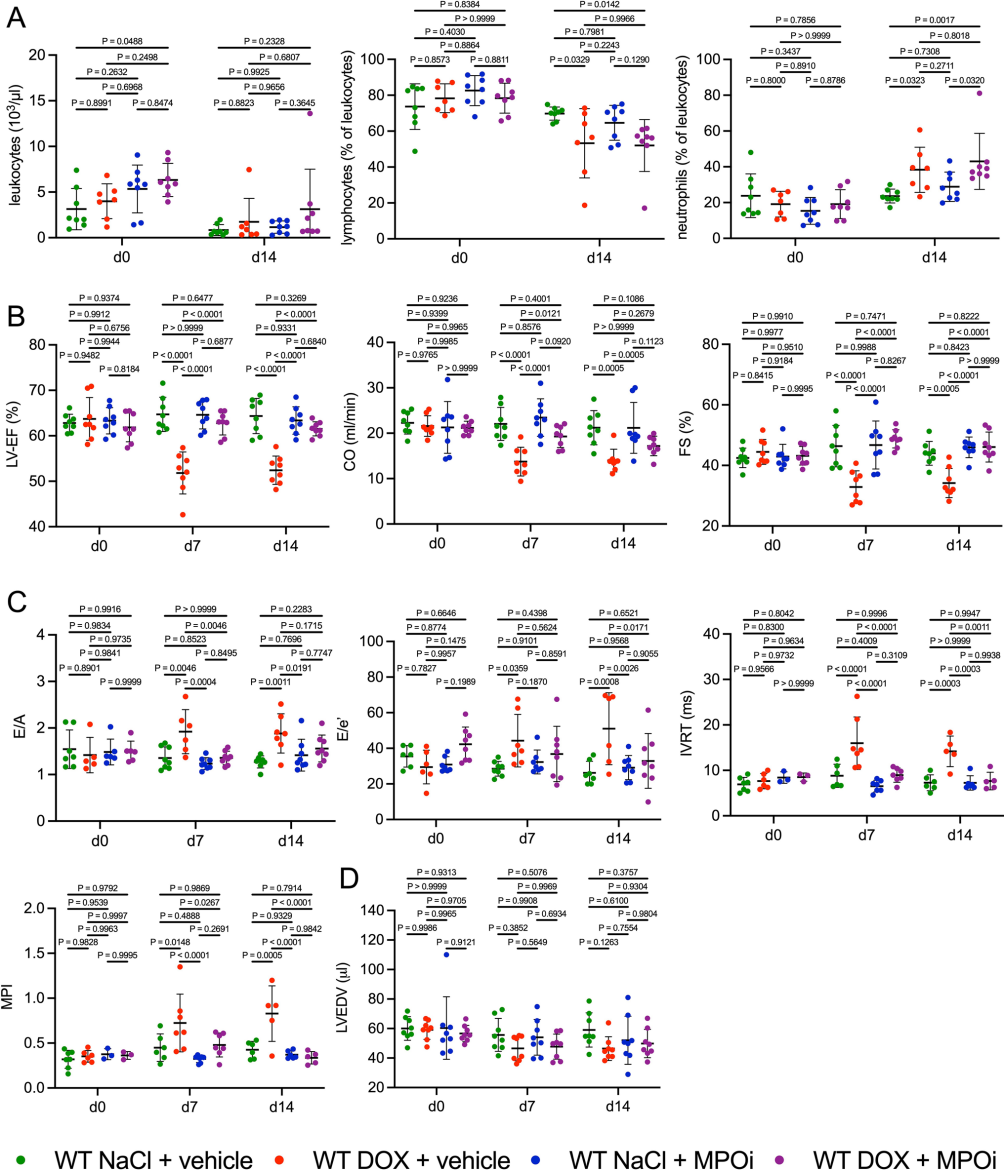
MPO inhibition attenuates acute AICM. **A** Blood leukocyte (10^3^/µl), lymphocyte (% of leukocytes), and neutrophil levels (% of leukocytes). d = day. n = 7–8 per group. **B** Echocardiographic markers of systolic LV function: LV ejection fraction (LV-EF; %), cardiac output (CO; ml/min), fractional shortening (FS; %). n = 8 per group. **C** Markers of diastolic LV function: E/A, E/e’, isovolumetric relaxation time (IVRT, ms), myocardial performance index (MPI). n = 3–8 per group (some values were missing since measurements could not be obtained due to low image quality). **D** LVEDV (µl). n = 8 per group. MPOi = MPO inhibitor 4-Aminobenzoic acid hydrazide. Vehicle = 10% DMSO dissolved in NaCl. Data are expressed as mean ± SD. Statistical significance was determined by two-way repeated measures ANOVA with Tukey’s multiple comparisons test

We eventually evaluated the therapeutic potential of pharmacological MPO inhibition in AICM. To determine persistency of DOX-related cardiac dysfunction and the effects of MPO inhibition, the observational period was extended to 14 days. DOX-treated mice still had lower systemic lymphocyte and higher neutrophil frequencies compared to NaCl-treated controls, whereas total leukocyte counts and frequencies of monocytes, eosinophils, or basophils did not differ between the treatment groups and genotypes (Figs. 8a and S9). Systolic and diastolic dysfunction was still present in DOX-treated WT (to the same extent observed after 7 days) but not in mice co-treated with the MPO inhibitor 4-ABAH (Fig. 8b, c). Differences in LV volume were not detected 7 or 14 days after treatment (Fig. 8d). In summary, these data demonstrate that pharmacological MPO inhibition prevents systolic dysfunction after exposure to DOX.

### MPO deficiency does not impair the anticancer efficacy of anthracyclines

To determine the impact of MPO deficiency on the anticancer efficacy of DOX, we utilized an anthracycline-responsive Burkitt Lymphoma model (Fig. S10A). A single intravenous dose of DOX, which was administered 7 days after tumour cell implantation, significantly increased survival in WT mice. DOX-treated WT and *Mpo*^*−*/*−*^ mice did not display significant differences in survival. Interestingly, a non-significant 10% increase in median survival alongside a significant reduction in weight loss prior to death was observed in DOX-treated *Mpo*^*−*/*−*^ compared to WT mice (median survival 33 vs. 30 days, *P* = 0.1, Fig. S10B, C). Collectively, these data clearly demonstrate that MPO deficiency does not negatively impact the anticancer efficacy of DOX.

## Discussion

Elevated plasma MPO levels are associated with an increased risk of cardiotoxicity in DOX-treated cancer patients [29, 45]. The present study provided first evidence for a causal link between MPO and AICM. Therefore, MPO emerges as a promising therapeutic target for prevention of AICM.

Considering that intraperitoneal injections of anthracyclines are associated with high mortality and may cause systemic inflammation due to gut damage and endotoxin leakage [9, 12, 56], we utilized a murine model of AICM in which DOX was administered via a jugular vein catheter. We showed that DOX directly induced MPO release by neutrophil-like HL60 cells in vitro and detected cardiac neutrophil infiltration and increased MPO levels in mice injected with DOX. Cardiac neutrophil infiltration after DOX injection was attenuated in *Mpo*^−/−^ mice. We have shown that MPO facilitates neutrophil recruitment by its positive surface charge [27], which might explain why *Mpo*^−/−^ mice had lower cardiac neutrophil levels, and obtained consistent findings in models of myocardial ischemia [36].

Genetic ablation and pharmacological inhibition of MPO protected mice from AICM. These findings confirm recent work by Sano et al., who observed cardiac neutrophil infiltration in C57BL/6J mice at the same time (1 week) after a single injection of DOX [48] and revealed that antibody-mediated neutrophil-depletion (anti-Ly6G) or neutrophil recruitment-inhibition (anti-CXCR2) prevented DOX-related cardiotoxicity [48]. Our study is an important extension of these findings since (1) we mechanistically unravel the role of MPO in causing AICM and (2) pharmacological MPO inhibition—unlike neutrophil depletion—represents a clinically feasible treatment strategy. Proteomics of cardiac tissue identified DOX-related upregulation of pathways associated with oxidative stress response, inflammation, fibrosis, and cell death. Furthermore, DOX-treated MPO-deficient mice were predicted to exhibit downregulation of oxidative stress response-, and inflammation-related pathways compared to WT animals. Downstream analyses largely confirmed these findings. Proteomics overall detected only minor differences in cardiac protein expression between DOX-treated wildtype and MPO-deficient mice. Potential reasons include but are not limited to (1) low sensitivity of proteomics to detect proteins with low abundance in cardiac tissue due to high expression of few structural/contractile proteins [30] and (2) a predominant role of MPO in mediating oxidative modifications rather than direct changes in protein expression. Accordingly, our data indicates that MPO-deficient mice were protected from DOX-related carbonylation of myofibrillar proteins, a well-known mechanism of cardiac contractile dysfunction [6]. In vitro experiments provided further evidence for a role of MPO in mediating DOX-related impairment of cardiomyocyte contractility: hiPSC-CMs co-treated with DOX and MPO exhibited a marked and sustained reduction in contractility compared to treatment with DOX alone, which was attenuated by pharmacological MPO inhibition. Additionally, MPO-treated hiPSC-CMs displayed a reduction in beating rate. While oxidative stress was recently implicating in mediating bradyarrhythmia [10], potential targets of MPO contributing to the observed reduction in beating rate remain to be investigated in future studies. DOX treatment was associated with increased cardiac expression of NOX2, which has been implicated in anthracycline-related ROS-formation [63], irrespective of the genotype. Cardiac expression of *Nox1*, *Nox3, Xo, Nos1*, and *Nos2* were neither impacted by DOX nor by MPO deficiency. In other words, the observed differences in oxidative stress between WT and *Mpo*^−/−^ mice were independent of ROS-formation by NOX1-3, NOS1-2, or XO. Interestingly, we revealed elevated *Nos3* mRNA levels in DOX-treated *Mpo*^−/−^ versus WT hearts. Considering the conflicting reports on the role of NOS3 in either promoting [39] or mitigating [61] AICM, exploring the pathophysiological implications of this observation emerges as a compelling avenue for future research.

Proteomics suggested a DOX-related increase in cell death that could be confirmed by immunoblots and histological analysis. Particularly, we revealed enhanced expression of Cleaved Caspase 3, and a higher count of apoptotic (TUNEL^+^) cells in cardiac tissue of DOX-treated WT that was attenuated in *Mpo*^−/−^ mice. Furthermore, phosphorylation of p38 MAPK was increased in cardiac tissue of DOX-treated WT versus *Mpo*^−/−^ mice and NaCl-treated controls. p38 MAPK, which has been shown to promote cardiomyocyte apoptosis, is activated by pro-inflammatory cytokines and ROS [13]. Of note, HOCl, the enzymatic product of MPO, is a potent activator of p38 MAPK [35, 36]. Our data suggests that MPO-dependent activation of p38 MAPK is critically involved in mediating DOX-related cardiomyocyte apoptosis, an established mechanism of AICM [11].

In line with previous reports [50, 62], we observed cardiac macrophage infiltration and increased cardiac expression of pro-inflammatory cytokines (Il1-β, and Tnf-α), chemokines (Cxcl1), and markers of inflammatory endothelial activation (Icam-1*)* in DOX- versus NaCl-treated WT. MPO deficiency attenuated DOX-related cardiac inflammation. This finding is consistent with data from our group and others indicating that MPO electrostatically facilitates leukocyte recruitment [27], whereas MPO inhibition/depletion attenuates cardiac macrophage recruitment after myocardial infarction [2, 37]. The course of events (DOX-related cardiac injury triggering MPO-dependent inflammation versus DOX-related neutrophil activation directly inducing cardiac inflammation) cannot be certainly determined. Nevertheless, given the significance of pro-inflammatory immunity in mediating cardiac dysfunction [1], it is conceivable that the observed cardioprotection in *Mpo*^−/−^ mice might be linked, at least in part, to the alleviation of cardiac inflammation. Despite clear signs of LV dysfunction, neither LV dilation nor cardiac hypertrophy were detectable in our model of AICM. DOX-treatment was associated with early signs of cardiac fibrosis in the perivascular area, but interstitial fibrosis and expression of pro-fibrotic genes were unaffected in DOX- versus NaCl-treated mice. These data argue against a prominent role of cardiac remodelling in early DOX-related cardiotoxicity and indicate that impaired sarcomere function, increased apoptosis and inflammation primarily contributed to the observed phenotype. Nevertheless, cardiac remodelling and fibrosis could become an important disease-mediating mechanism upon repetitive exposure to DOX in the long-term, as previously reported [18].

A limitation of our study is that it focusses solely on acute AICM and does not incorporate a chronic model of DOX-related cardiotoxicity. It is noteworthy that we were not successful in generating a pronounced cardiac phenotype by chronic administration of DOX. While repeated tail vein injections led to the development of tail necrosis, animals subjected to repeated i.p. injections (5 mg/kg bodyweight DOX weekly for up to 7 weeks) did not develop significant cardiotoxicity. This stands in contrast to previous studies that achieved the establishment of chronic AICM by repeated i.p. injections. One explanation may lie in the distinct genetic backgrounds of the utilized strains, as several studies have consistently demonstrated that lineage-specific effects determine the susceptibility of experimental animal models to cardiotoxicity as well as cardioprotection [3, 22, 25, 26]. Nonetheless, both acute models, utilizing a single high dose, and chronic models, employing repetitive injections of small doses, have been widely used to study AICM in mice [44]. Several considerations supported our decision to employ an acute model of AICM. Firstly, early cardiotoxicity represents the primary manifestation of AICM in patients, as evidenced by a recent study indicating that the median time to onset of AICM was 3.5 months, with 98% of cases occurring within the first year after treatment [7]. In mice, this time period roughly corresponds to 9 days [17]. Furthermore, cardiac neutrophil infiltration has been reported to peak 1 week after administration of DOX, making it a relevant timepoint to investigate the role of MPO in AICM [48].

Dexrazoxane, the only approved compound for prevention of AICM [8, 42], is an EDTA derivate that chelates DOX-complexed iron ions, thereby preventing superoxide formation. This mechanism supports the importance of ROS production by iron containing enzymes such as MPO in AICM [28]. Since iron chelating MPO inhibitors have been scarcely investigated [38], investigating the impact of dexrazoxane on MPO emerges as an interesting topic for future research. Despite its proven capability to prevent AICM, clinical application of dexrazoxane has been restricted since concerns were raised that it might reduce the anti-tumour efficacy of anthracyclines [8, 42]. Utilizing a murine model of anthracycline-sensitive Burkitt Lymphoma, we demonstrated that MPO deficiency does not impair the anticancer efficacy of DOX. Rather, we revealed a tendency towards better outcomes in DOX-treated *Mpo*^−/−^ versus WT mice, which is in line with previous work showing that MPO deficiency limits tumour growth [47].

In conclusion, our study provides evidence that MPO is causally involved in pathogenesis of AICM. The availability of oral MPO inhibitors (e.g. AZD4831), which have been proven to be safe and efficient in humans [40], and the possibility to identify patients which might particularly benefit from MPO inhibition by measuring plasma MPO levels, suggest that such therapy harbors considerable translational potential. Given the lack of approved pharmacotherapies for prevention of AICM, MPO inhibition emerges as a promising treatment strategy that warrants further investigation.

### Supplementary Information

Below is the link to the electronic supplementary material.

Supplementary file 1 (DOCX 5197 KB)

Supplementary file 2 (XLSX 451 KB)

## Data Availability

The mass spectrometry proteomics data have been deposited to the ProteomeXchange Consortium via the PRIDE [43] partner repository with the dataset identifier PXD037524. Other data underlying this article will be shared on reasonable request to the corresponding authors.
