# Supplementary material for: Myeloperoxidase is a critical mediator of anthracycline-induced cardiomyopathy
Source: Basic Res Cardiol. 2023 Sep 1;118(1):36. doi: 10.1007/s00395-023-01006-0 (PMC10474188; doi:10.1007/s00395-023-01006-0)
Supplement: Supplementary file 1 — (DOCX 5197 KB) [file 395_2023_1006_MOESM1_ESM.docx]

**Supplement`ary Material**

**Article title: Myeloperoxidase is a Critical Mediator of Anthracycline-induced Cardiomyopathy**

**Journal Name: Basic Research in Cardiology**

**Authors and affiliations:**

**Felix Sebastian Nettersheim^1,2*^, Johannes David Schlüter^1,2*^, Wiebke Kreuzberg^1,2^, Dennis Mehrkens^1,2^, Simon Grimm^1,2^, Harshal Nemade^1,2^, Simon Braumann^1,2^, Alexander Hof^1,2^, Henning Guthoff^1,2^, Vera Peters^1,2^, Friedrich Felix Hoyer^1,2^, Yulia Kargapolova^1,2^, Jan-Wilm Lackmann^3^, Stefan Müller^2^, Christian P. Pallasch^4,5^, Michael Hallek^4,5^, Agapios Sachinidis^2,6^, Matti Adam^1,2^,** **Holger Winkels^1^, Stephan Baldus^1,2^, Simon Geißen^1,2†^, Martin Mollenhauer^1,2†^**

*^1^ Department of Cardiology, Faculty of Medicine and University Hospital Cologne, University of Cologne, Cologne, Germany*

*^2^ Center for Molecular Medicine Cologne (CMMC), University of Cologne, Cologne, Germany*

*^3^CECAD, University of Cologne, Faculty of Mathematics and Natural Sciences, Cologne, Germany*

*^4^CECAD, University of Cologne, Faculty of Medicine and University Hospital Cologne, Cologne, Germany*

*^5^ Department I of Internal Medicine, Center for Integrated Oncology (CIO) Köln-Bonn*

*^6^Institute of Neurophysiology, Faculty of Medicine, University of Cologne, Cologne, Germany.*

**^,^***^†^***These authors contributed equally to the manuscript.*

Corresponding authors:

Felix Nettersheim, Department of Cardiology, University Hospital Cologne, Kerpener Str. 62, 50937 Cologne, Germany, Email: felix.nettersheim@uk-koeln.de

Martin Mollenhauer, Department of Cardiology, University Hospital Cologne, Kerpener Str. 62, 50937 Cologne, Germany, Email: martin.mollenhauer@uk-koeln.de

1. **Supplementary Methods**
   1. **Echocardiography**

Transthoracic echocardiography was performed before DOX/NaCl injections (baseline) and at the indicated timepoints thereafter (7 days post injection and, additionally, 14 days post injection in the MPO inhibitor experiment) in isoflurane-anaesthetized mice (Isofluran-Piramal®, Piramal Critical Care, Voorschoten, The Netherlands; 5 % vol/vol for induction and 2 % vol/vol for maintenance of anaesthesia). Parasternal long axis (PSLAX) and apical four-chamber (A4C) views of the heart were imaged in B-Mode, M-Mode, and electrocardiogram-triggered kilohertz visualization (EKV) using a Vevo 3100 ultrasound system (FUJIFILM VisualSonics, Toronto, ON, Canada) equipped with a MX550D transducer (25-55 MHz, centre transmit: 40 MHz, axial resolution: 40 μm) by a blinded examiner. To determine diastolic function, pulsed wave (PW) doppler recordings of the mitral inflow and tissue doppler (TD) recordings of the septal mitral anulus were obtained from the apical four-chamber view. Measurements were analysed using Vevo Lab v5 (VisualSonics, Fujifilm, Tokio, Japan) by two blinded investigators. Left ventricular ejection fraction (LV-EF) and cardiac output (CO) were determined by planimetry of end-systolic (LVESV) and end-diastolic (LVEDV left ventricular volumes in the B-mode/EKV PSLAX view. Fractional shortening (FS) was obtained by measuring end-systolic and end-diastolic left ventricular diameters in the M-mode PSLAX view. E and A waves, isovolumetric relaxation time (IVRT) and MPI (myocardial performance index: [IVRT + isovolumetric contraction time]/ejection time) were obtained from mitral inflow PW recordings and e’ waves from mitral annular TD recordings. Mice with substantially impaired left ventricular systolic function (LVEF < 50%) at baseline were excluded from the study.

- 1. **Tissue preparation**

Atria and the right ventricle were removed and the left ventricle (LV) was dissected into 3 longitudinal pieces. The first piece of the LV was either embedded in Tissue-Tek optimum cutting temperature (O.C.T.) compound and frozen on dry ice or fixed in 3.7% formaldehyde solution for 2 days and subsequently embedded in paraffin for histological analyses. Serial sections of tissue specimen with 5 µm thickness were prepared and mounted in groups of three on a microscope slide. The other two pieces of the LV were snap-frozen in liquid nitrogen for further molecular biological analyses.

- 1. **Immunofluorescence stainings**

Frozen sections were thawed, fixed with 4% paraformaldehyde solution for 10 minutes, and blocked for 60 minutes with blocking solution (3-5 % rat serum or fetal calf serum plus 1 % BSA in 1 x PBS). Samples were then incubated with primary antibodies (**Supplementary Table 1**; staining buffer: 1 x PBS containing 1.5 % blocking solution) overnight at 4 °C in a dark humid chamber. Samples stained with an anti-3-nitrotyrosine primary antibody were subsequently incubated with a secondary antibody (Alexa Fluor 594 conjugated goat anti-rabbit, Invitrogen A-11012, 1/100, solved in 1 x PBS) for 60 minutes at room temperature (RT). The other primary antibodies (Ly6G, CD68, and F4/80) were pre-conjugated with Alexa Fluor 594 or APC. Finally, samples were stained with DAPI (1/1000, solved in 1 x PBS) for 15 minutes. Isotype controls were performed to confirm specificity of the primary antibodies. Images were acquired on a Keyence BZ-X800 microscope at 40 x magnification and the number of positively stained cells per visual field (Ly6G, F4/80, CD68) or the positively stained area (3-nitrotyrosine) were quantified by two blinded investigators using the Keyence BZ-II analyser software.

- 1. **TUNEL staining**

The Click-iT™ Plus TUNEL Assay Kit (Invitrogen, Waltham, MA, USA) was used for detection of apoptotic cells in frozen cardiac sections. Frozen sections were thawed, fixed, permeabilized and incubated with the assay solutions as indicated in the user guide. Finally, samples were stained with DAPI (1/1000, solved in 1 x PBS) for 15 minutes. Images were acquired on a Keyence BZ-X800 microscope at 40 x magnification and the number of positively stained cells per visual field was quantified by two blinded investigators using the Keyence BZ-II analyser software.

- 1. **Picrosirius red staining**

Paraffin-embedded slices were dewaxed, rehydrated and stained with PicroSirius Red solution (Merck KGaA, Darmstadt, Deutschland) for one hour in a humid chamber. Subsequently, slices were incubated two times with acetic acid solution for 30 seconds, dehydrated and mounted. Images were acquired on a on a Keyence BZ-X800 (Keyence, Osaka, Japan) microscope at 40 x magnification. Perivascular and interstitial fibrotic areas (red signal, % of visual field) were planimetrically quantified using BZ-X800 Analyser and Fiji ImageJ.

- 1. **Proteomics**
     1. **Tissue preparation and protein digestion**

Frozen cardiac tissue samples were homogenized in Precellys ceramic kit tubes (1.4 mm, 2.0 ml) containing 200 µl ice-cold Urea lysis buffer (8M Urea in 50mM Triethylammoniumbicarbonate [TEAB]) supplemented with 50x Protease Inhibitor cocktail (Roche, Mannheim, Germany) using Precellys 24 tissue homogenizer (Bertin Instruments, Montigny-le-Bretonneux, France). Supernatants were collected after repetitive vortexing and centrifugation, incubated with 25 Units Benzonase HC for 30 minutes at 37°C to degrade nucleic acids and centrifuged again. Pierce 660nm Protein Assay Kit (Thermo Scientific, Waltham, MA, USA) was used to determine protein concentrations.

50 µg of protein per sample were transferred to a 1.5 ml tube and incubated with Dithiothreitol (final concentration of 5 mM) for 1 hour at RT. Next, samples were incubated with Chloroacetamide at a final concentration of 40 mM for 30 minutes at RT in the dark and, subsequently, with Lys-C protease at an enzyme:substrate ratio of 1:75 for 4 hours at RT. Samples were diluted with 50 mM TEAB to achieve a final concentration of Urea ≤ 2 M and incubated with Trypsin at an enzyme:substrate ratio of 1:75 overnight at RT. The next day, formic acid at a final concentration of 1% was added to stop enzymatic digestion and peptides were purified with SDB-RPS (styrenedivinylbenzene reverse phase sulfonate) polymer sorbent StageTips (CDS Analytical, Oxford, PA, USA).

- - 1. **LCMS Data Independent Acquisition**

Samples were analyzed by the CECAD Proteomics Facility on an Orbitrap Exploris 480 (Thermo Scientific, granted by the German Research Foundation under INST 1856/71-1 FUGG) mass spectrometer equipped with a FAIMSpro differential ion mobility device that was coupled to an UltiMate 3000 (Thermo Scientific). Samples were loaded onto a precolumn (Acclaim 5 µm PepMap 300 µm Cartridge, Thermo Scientific) for 1 min at 15 µl flow before reverse-flushed onto an in-house packed analytical column (30 cm length, 75 µm inner diameter, filled with 2.7 µm Poroshell EC120 C18, Agilent, Santa Clara, CA, USA). Peptides were chromatographically separated at a constant flow rate of 300 nL/min and the following gradient: initial 6% B (0.1% formic acid in 80 % acetonitrile), up to 32% B in 72 min, up to 55% B within 7.0 min and up to 95% solvent B within 2.0 min, followed by column wash with 95% solvent B and reequilibration to initial condition. The FAIMS pro was operated at -50V compensation voltage and electrode temperatures of 99.5 °C for the inner and 85 °C for the outer electrode.

- - 1. **Spectrum library generation by Gas phase fractionation**

Aliquots from each sample were pooled and the pool was used for spectrum library generation by narrow window DIA of six 100 m/z gas phase fractions (GPF) covering the range from 400 m/z to 1000 m/z [9]. The Orbitrap was operated in DIA mode. MS1 scans of the respective 100 m/z gas phase fraction were acquired at 60k resolution. Maximum injection time was set to 60 ms and the AGC target to 100%. MS2 scans of the corresponding 100 m/z region were acquired in 24 x 4 m/z staggered windows resulting in 48 nominal 2 m/z windows after demultiplexing. MS2 settings were 30 k resolution, 60 ms maximum injection time and an AGC target of 100%. All scans were stored as centroid.

- - 1. **Data independent acquisition of samples**

MS1 scans were acquired from 390 m/z to 1010 m/z at 15k resolution. Maximum injection time was set to 22 ms and the AGC target to 100%. MS2 scans ranged from 400 m/z to 1000 m/z and were acquired at 15 k resolution with a maximum injection time of 22 ms and an AGC target of 100%. DIA scans covering the precursor range from 400 - 1000 m/z and were acquired in 75 x 8 m/z staggered windows, resulting in effective 4 m/z windows after deconvolution. All scans were stored as centroid.

- - 1. **Data processing**

Thermo raw files were demultiplexed and transformed to mzML files using the msconvert module in Proteowizard. MzML files were converted to dia file format in DIA-NN 1.8

- - 1. **Spectral Library**

A Mouse canonical Swissprot fasta file was converted to a Prosit upload file with the convert tool in encyclopedia 0.9.0 [8] using default settings: Trypsin, up to 1 missed cleavage, range 396 m/z – 1004 m/z, charge states 2+ and 3+, default charge state 3 and NCE 33. The csv file was uploaded to the Prosit webserver and converted to a spectrum library in generic text format [4]. The resulting library (16998 protein isoforms, 21698 protein groups and 1404872 precursors) was searched in DIA-NN 1.8 [3] with the 6 GPF runs to generate a project specific library (7177 protein isoforms, 7546 protein groups and 45716 precursors). The applied settings were: Output will be filtered at 0.01 FDR, N-terminal methionine excision enabled, maximum number of missed cleavages set to 1, min peptide length set to 7, max peptide length set to 30, min precursor m/z set to 400, Max precursor m/z set to 1000, cysteine carbamidomethylation enabled as a fixed modification.

- - 1. **Samples**

24 sample files were searched with DIA-NN 1.8 and the project library. In addition to the settings used for library generation, Rt dependend normalization and relaxed protein inference was used. Results were filtered on library q-value, protein group q-value, and global q-value of 0.01 or lower prior to MaxLFQ calculations using the DIA-NN R-package. Further data processing was performed in Perseus 1.6.15 [10].

- - 1. **Statistical analysis of proteomics data**

Differentially expressed (DE) proteins were determined by one-way analysis of variance (ANOVA) using Perseus 1.6.15. A False Discovery Rate (Benjamini-Hochberg FDR) adjusted value of q < 0.05 was considered statistically significant. Statistical significance of between-group differences of DE proteins were determined with post-hoc Tukey’s test (P < 0.05) using GraphPad Prism 9. Principal component analysis was performed using Perseus 1.6.15 (number of components: 5).

- - 1. **Pathway enrichment analysis**

Metascape (<http://metascape.org>, default parameters) [11] was used for pathway enrichment analysis. To determine directional regulation of pathways either up- or downregulated proteins were used as input for Metascape analysis.

- 1. **Immunoblot analysis**

Frozen cardiac tissue samples were homogenized in Precellys ceramic kit tubes (1.4 mm, 2.0 ml) containing 200 µl ice-cold RIPA lysis buffer (150 mM NaCl, 5 mM EDTA pH 8.0, 50 mM Tris pH 8.0, 1 % NP40, 0.5 % sodium deoxycholate 0.1 % SDS) supplemented with protease and phosphatase inhibitors (Roche, Mannheim, Germany) using Precellys 24 tissue homogenizer. Supernatants were collected after repetitive vortexing and centrifugation. Pierce BCA Protein Assay Kit (Thermo Scientific) was used to determine protein concentrations. 15 µg of protein from each sample were separated under reducing conditions on SDS–polyacrylamide gels and transferred to nitrocellulose membranes. Sufficient transfer of proteins was verified by Ponceau staining. Membranes were blocked for one hour at RT, incubated with primary antibodies overnight at 4 °C and, finally, incubated with secondary antibody for one hour at RT. Proteins were detected with the primary antibodies listed in **Table S1**. Horseradish peroxidase-conjugated goat anti-rabbit IgG (1/10000, Vector Laboratories #PI-1000) was used as a secondary antibody. After incubation with SuperSignal West Femto Maximum Sensitivity Substrate (Thermo Fisher Scientific) and Amersham ECL Western Blotting Detection Reagents (GE Healthcare, Buckinghamshire, UK), the fluorescent signal was visualised with a Fusion FX imaging system (Vilber Lourmat, Collegien, France). Bands were quantified using Fusion FX software. Uncropped immunoblots are shown in in **Fig. S11 and S12**.

- 1. **Protein carbonyl assay**

Frozen cardiac tissue samples were homogenized in Precellys ceramic kit tubes (1.4 mm, 2.0 ml) containing 200 µl ultrapure water using Precellys 24 tissue homogenizer. Supernatants were collected after repetitive vortexing and centrifugation. Protein concentrations were determined by Pierce BCA assay (Thermo Scientific) and samples were diluted to the lowest detected concentration (139 µg/100 µl). 100 µl of samples were finally analysed using Protein Carbonyl Content Assay Kit (MAK094, Sigma-Aldrich, St. Louis, MO, USA) according to the manufacturer’s instructions.

- 1. **Protein Carbonyl Immunoblot**

Abcam Protein Carbonyl Assay Kit (ab178020, Cambridge, UK) was used to measure protein carbonylation by immunoblots. Frozen cardiac tissue samples were homogenized in Precellys ceramic kit tubes (1.4 mm, 2.0 ml) containing 50 µl extraction buffer (contained in the kit) using Precellys 24 tissue homogenizer. Supernatants were collected after repetitive vortexing and centrifugation. Protein concentrations were determined using the Pierce 660nm Protein Assay Kit (Thermo Scientific) and samples were diluted to a protein concentration of 3 mg/ml. 2,4-dinitrophenylhydrazone (DNP-hydrazone) derivatization was performed as described in the protocol booklet. 7.5 µg of derivatized protein from each sample were separated on SDS–polyacrylamide gels and transferred to nitrocellulose membranes. Ponceau S staining was performed for protein normalization. Membranes were blocked for one hour at RT, incubated with primary anti-DNP antibody (contained in the kit) overnight at 4 °C and, finally, incubated with secondary antibody (horseradish peroxidase-conjugated goat anti-rabbit IgG, 1/10000, Vector Laboratories #PI-1000) for one hour at RT. After incubation with SuperSignal West Femto Maximum Sensitivity Substrate (Thermo Fisher Scientific) and Amersham ECL Western Blotting Detection Reagents (GE Healthcare, Buckinghamshire, UK), the fluorescent signal was visualised with a Fusion FX imaging system (Vilber Lourmat, Collegien, France). Bands were quantified using Fusion FX software. The carbonylation index was calculated as the ratio of anti-DNP-stained bands (carbonylated protein) and corresponding bands in the Ponceau S staining (total protein). To identify carbonylated proteins, 7.5 µg protein of the same samples (one gel) used in the anti-DNP immunoblot were separated on SDS–polyacrylamide gels and subjected to immunoblot analysis as described above. Recent evidence suggested that 𝛼-sarcomeric Actin (𝛼-SCA), tropomyosin [2] and myosin-heavy-chain (MHC) [1] are particularly prone to carbonylation. Staining of immunoblots with anti-𝛼-SCA and anti-MHC antibodies yielded bands of the same molecular size as the two most prominent anti-DNP-stained bands. No bands at the molecular size of tropomyosin could be detected upon anti-DNP staining. Thus, these data are consistent with previous reports that protein oxidation in cardiac tissue predominantly affects 𝛼-SCA and MHC. Uncropped immunoblots are shown in **Fig. S13**.

- 1. **Xanthine Oxidase Activity Assay Kit**

Cardiac tissue samples were homogenized and Pierce BCA Protein Assay Kit (Thermo Scientific) was used to determine protein concentrations as described in **1.7**. To assess Xanthine oxidase activity Abcam Xanthine Oxidase Activity Assay Kit (ab102522) was used according to manufacturer's instructions.

- 1. **Real-time quantitative PCR**

Total RNA from cardiac tissue samples was extracted and purified using the RNeasy Mini kit (Qiagen, Venlo, The Netherlands). The NanoDrop 2000 Spectrophotometer (Thermo Fisher Scientific) was used to measure RNA concentrations. Complimentary DNA (cDNA) was reverse transcribed using the High-Capacity cDNA Reverse Transcription Kit (Applied Biosystems, Waltham, MA, USA) from 1 μg RNA. Real-time quantitative PCR (qPCR) was performed on a QuantStudio^TM^ 3 Real-Time PCR System (Thermo Fisher, Rockford, USA) using GoTaq qPCR Master Mix (Promega, Madison, WI, USA). Primer sequences are provided in **Table S2**. The relative mRNA expression (fold change relative to WT) was quantified using the 2^-∆∆CT^ method (β-Actin or GAPDH were used for normalization).

- 1. **ELISA-based protein quantification**

Frozen cardiac tissue samples were homogenized and protein concentrations were analysed as described in 1.7. Samples were diluted to the lowest detected concentration. Blood samples were obtained from deeply anesthetized mice by cardiac puncture, collected in heparinized tubes and plasma was isolated by centrifugation for 10 min at 1300 x g. MPO, IL-1β, TNF-𝛼, and Troponin I were measured using the following ELISA kits according to the manufacturer’s instructions: Mouse MPO ELISA kit (HK210, Hycult Biotech, Plymouth Meeting, PA, USA), Mouse IL-1 beta SimpleStep ELISA Kit (ab197742, Abcam, Cambridge, UK), Mouse TNF-alpha Quantikine ELISA Kit (MTA00B, R&D Systems, Minneapolis, MN, USA), and Mouse Cardiac Troponin I ELISA Assay Kit (SEA478Mu, Cloud-Clone, Katy, TX, USA),

- 1. **Haematology analysis**

Blood samples were obtained from deeply anesthetized mice by puncture of the Vena facialis (day 0) or by cardiac puncture (day7/14), collected in heparinized tubes, and analysed with the Element HT5 haematology analyser (HESKA, Loveland, Colorado, USA).

- 1. **Plasma cytokine array**

Blood samples were obtained from deeply anesthetized mice by cardiac puncture, collected in heparinized tubes and plasma was isolated by centrifugation for 10 min at 1300 x g. 100 µl plasma from two mice was pooled and subjected to Abcam Mouse Cytokine Antibody Array (ab133993) according to manufacturer’s instructions. Spot intensities were quantified using Protein Array Analyzer for ImageJ.

- 1. **Cardiomyocyte cell culture and functional studies using the xCELLigence RTCA**

The xCELLigence RTCA Cardio system (Agilent, San Diego, CA, USA) was used to analyse the effects of DOX and/or MPO on beating profiles of human pluripotent stem cell-derived cardiomyocytes (hiPSC-CMs; iCell Cardiomyocytes®; Cellular Dynamics International, Madison, WI, USA). Functional studies were performed as reported previously [7]. In brief, the cryopreserved hiPSC-CMs were thawed and 25 × 10^3^ cells per well were seeded on a pre-equilibrated fibronectin-coated E-plate Cardio 96 (Agilent, San Diego, CA, USA) using iCell cardiomyocyte plating medium (iCell-PM, Cellular Dynamics International, Madison, WI, USA). 48 hours post seeding the media was changed to iCell cardiomyocyte Maintenance Medium (iCell-MM, Cellular Dynamics International, Madison, WI, USA) and the plate was inserted into the xCELLigence station kept inside of a standard cell culture incubator at 5% CO2, 37 °C. Treatments were started after stable beating profiles were observed in all wells. Cardiomyocytes were left untreated (Control), or exposed to any of the following treatment conditions: 1. H_2_O_2_ (40 µM) and MPO (10 µg/ml), 2. DOX (156 nM), 3. DOX (156 nM) and MPO (10 µg/ml), 4. DOX (156 nM) and MPO-inhibitor (MPOi) 4-Aminobenzoic acid hydrazide (4-ABAH; 50 µM), or 5. DOX (156 nM), MPO (10 µg/ml) and 4-ABAH (50 µM). The xCELLigence RTCA Cardio system utilizes impedance measurements to dynamically monitor the beating amplitude, representing cardiomyocyte contractility, the beating rate, and the cell index, a measure of cellular viability. Impedance measurements were recorded every 15 minutes for 48 hours. Data were acquired using RTCA Cardio software version 1.0 (ACEA Biosciences, Inc., San Diego, CA, USA). Measurements obtained within a 6-hour interval prior to treatment (baseline = 0h), and within 2-hour intervals thereafter (each 8 consecutive measurements) were averaged for graphical visualization and statistical analysis.

- 1. **HL60 cell culture**

Differentiated human promyelocytic HL60 cells were used as an *in vitro* model system to study the response of neutrophils to DOX treatment. The cells were differentiated into neutrophils using dimethyl sulfoxide (DMSO) as described previously[5] and subsequently treated with DOX. HL60 cells were cultured in RPMI 1640 medium containing 10% heat-inactivated FBS and 1% penicillin/streptavidin in humidified air containing 5% CO2 at 37°C until desired confluence. Subsequently, 1.25% DMSO was added to 5 x 10^5^ cells/mL. After 5 days of incubation, 5 x 10^5^ cells/mL (2 mL each) were seeded in 12-well plates and treated with DOX (156 nM, 300 nM, 600 nM) or LPS (100 ng/mL, positive control) for 2 h. After centrifugation (300 x g, 3 min), the cell supernatant was collected and stored at -80°C for further analysis.

- 1. **Tumour survival model**

All cell culture reagents were ordered from Gibco (Thermo Scientific) and all procedures were performed under sterile conditions. Transplantable *Eμ-myc* B-cell lymphoma cells [6] were maintained as suspension culture in BCM media composed of 50% IMDM and 50% DMEM supplemented with 10% FBS, 50µM β-mercaptoethanol, 1x GlutaMAX and 1x Pen/Strep. Media was changed every 2/3 days depending on the cell density. Cells were passaged at 70% confluence. For mice injections, the cells were collected, centrifuged and washed with HBSS. 1 X 10^7^ cell were resuspended in 150 µl HBSS and injected into male and female WT and *Mpo^-/-^* mice (10-14 weeks of age) via the tail vein. Seven days after tumour cell injection, a single bolus DOX (10 mg/kg bodyweight; dissolved in 0.9% saline at a concentration of 3 mg/ml) or 0.9% saline (NaCl; 6.67 ml/kg bodyweight) was administered via a jugular vein catheter as described in chapter 2.2 of the main manuscript. The mice were monitored until reaching the endpoint of the study by achieving the termination criteria according to the animal welfare guidelines or due to natural death.

- 1. **References**

1. Balogh Á, Santer D, Pásztor ET, Tóth A, Czuriga D, Podesser BK, Trescher K, Jaquet K, Erddi F, Édes I, Papp Z (2014) Myofilament protein carbonylation contributes to the contractile dysfunction in the infarcted LV region of mouse hearts. Cardiovasc Res 101:108–119. doi: 10.1093/CVR/CVT236

2. Canton M, Menazza S, Sheeran FL, Polverino De Laureto P, Di Lisa F, Pepe S (2011) Oxidation of Myofibrillar Proteins in Human Heart Failure. J Am Coll Cardiol 57:300–309. doi: 10.1016/J.JACC.2010.06.058

3. Demichev V, Messner CB, Vernardis SI, Lilley KS, Ralser M (2019) DIA-NN: neural networks and interference correction enable deep proteome coverage in high throughput. Nat Methods 2019 171 17:41–44. doi: 10.1038/s41592-019-0638-x

4. Gessulat S, Schmidt T, Zolg DP, Samaras P, Schnatbaum K, Zerweck J, Knaute T, Rechenberger J, Delanghe B, Huhmer A, Reimer U, Ehrlich HC, Aiche S, Kuster B, Wilhelm M (2019) Prosit: proteome-wide prediction of peptide tandem mass spectra by deep learning. Nat Methods 2019 166 16:509–518. doi: 10.1038/s41592-019-0426-7

5. Jacob C, Leport M, Szilagyi C, Allen JM, Bertrand C, Lagente V (2002) DMSO-treated HL60 cells: a model of neutrophil-like cells mainly expressing PDE4B subtype. Int Immunopharmacol 2:1647–1656. doi: 10.1016/S1567-5769(02)00141-8

6. Meacham CE, Ho EE, Dubrovsky E, Gertler FB, Hemann MT (2009) In vivo RNAi screening identifies regulators of actin dynamics as key determinants of lymphoma progression. Nat Genet 2009 4110 41:1133–1137. doi: 10.1038/ng.451

7. Nemade H, Chaudhari U, Acharya A, Hescheler J, Hengstler JG, Papadopoulos S, Sachinidis A (2018) Cell death mechanisms of the anti-cancer drug etoposide on human cardiomyocytes isolated from pluripotent stem cells. Arch Toxicol 92:1507–1524. doi: 10.1007/S00204-018-2170-7/FIGURES/7

8. Searle BC, Pino LK, Egertson JD, Ting YS, Lawrence RT, MacLean BX, Villén J, MacCoss MJ (2018) Chromatogram libraries improve peptide detection and quantification by data independent acquisition mass spectrometry. Nat Commun 2018 91 9:1–12. doi: 10.1038/s41467-018-07454-w

9. Searle BC, Swearingen KE, Barnes CA, Schmidt T, Gessulat S, Küster B, Wilhelm M (2020) Generating high quality libraries for DIA MS with empirically corrected peptide predictions. Nat Commun 2020 111 11:1–10. doi: 10.1038/s41467-020-15346-1

10. Tyanova S, Temu T, Sinitcyn P, Carlson A, Hein MY, Geiger T, Mann M, Cox J (2016) The Perseus computational platform for comprehensive analysis of (prote)omics data. Nat Methods 2016 139 13:731–740. doi: 10.1038/nmeth.3901

11. Zhou Y, Zhou B, Pache L, Chang M, Khodabakhshi AH, Tanaseichuk O, Benner C, Chanda SK (2019) Metascape provides a biologist-oriented resource for the analysis of systems-level datasets. Nat Commun 2019 101 10:1–10. doi: 10.1038/s41467-019-09234-6

1. **Supplementary Figures**


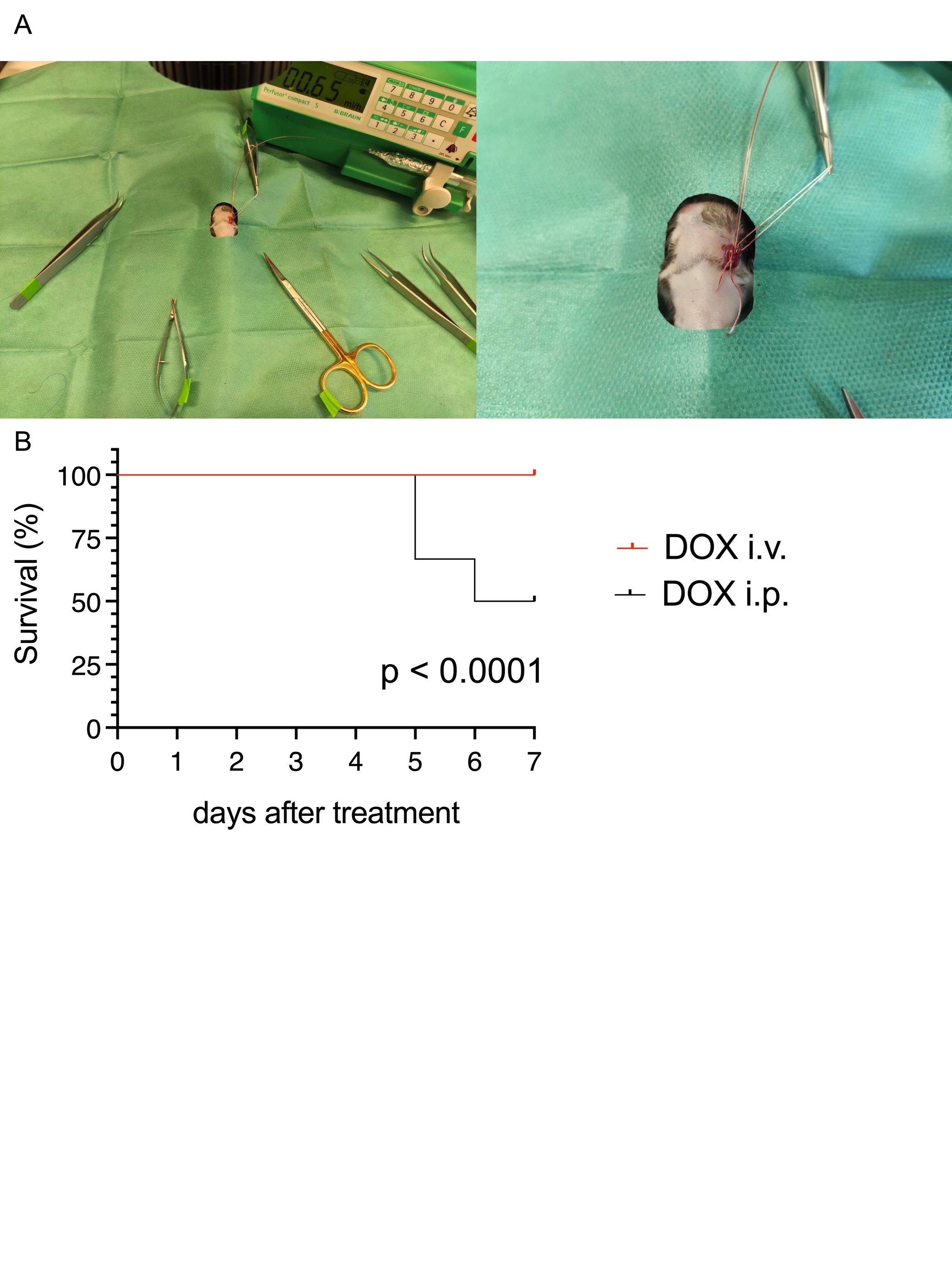


**Fig. S1** Murine model of anthracycline-induced cardiotoxicity. (A) Mice were deeply anesthetized with isoflurane and pre-procedural buprenorphine treatment. The left jugular vein was cleared, a small catheter was carefully introduced, and the catheter was fixed with a suture. DOX was slowly injected over 30 minutes. After withdrawing the catheter, the puncture site was closed with the same suture. Finally, the skin was stitched up. (B) Central-venous injection of DOX prevented premature mortality, which was observed after i.p. injection (n = 6 (i.p.) and 48 (i.v.)). Statistical significance was determined by log-rank (Mantel– Cox) test.

**
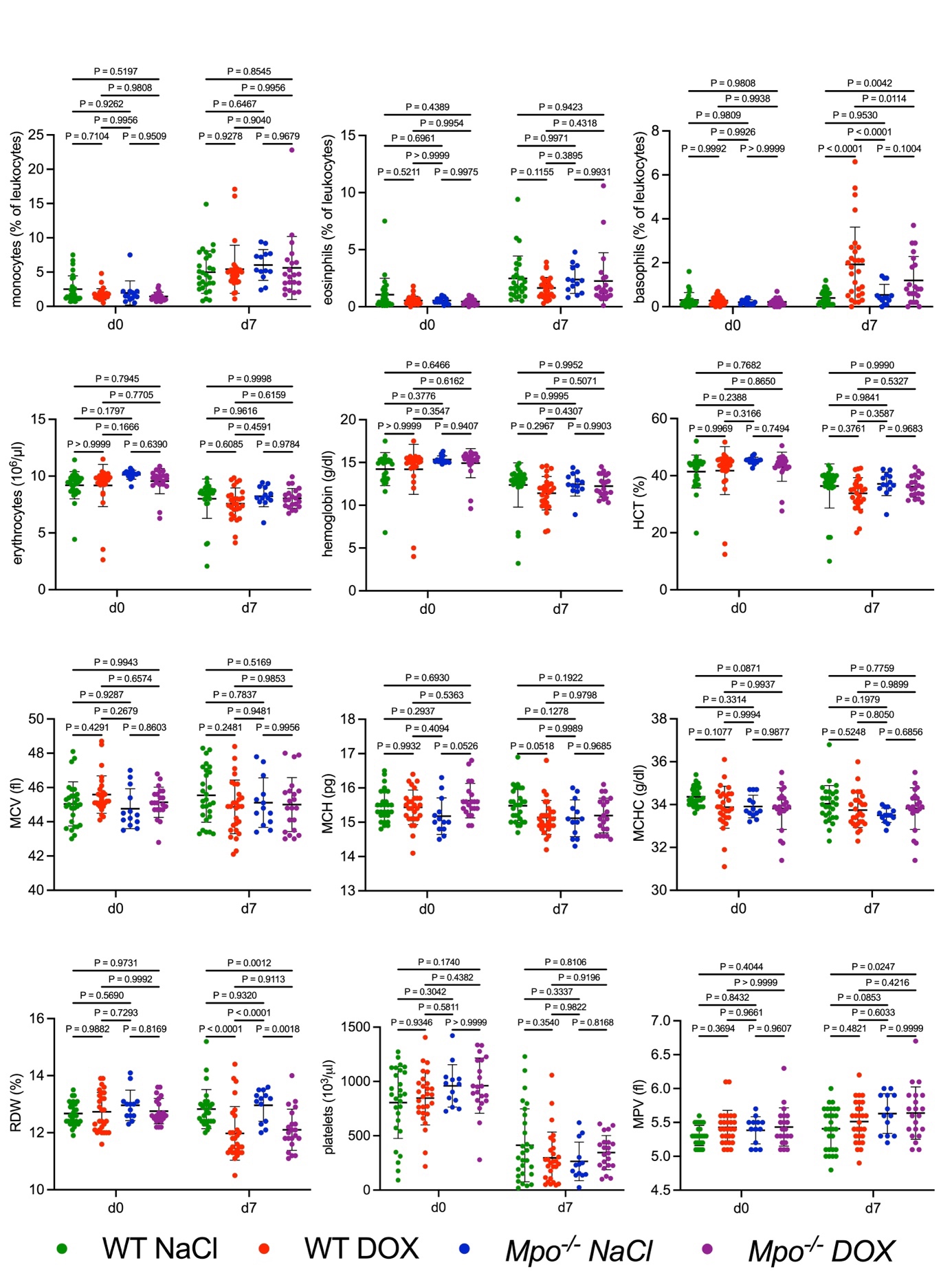
Fig. S2** Haematological parameters. Blood samples were taken before and seven days after treatment (d = day). HCT = haematocrit, MCV = mean corpuscular volume, MCH = mean corpuscular haemoglobin, MCHC = mean corpuscular haemoglobin concentration, RDW = red cell distribution width. Data are expressed as mean ± SD. n = 13-28 per group. Statistical significance was determined by two-way repeated measures ANOVA with Tukey’s multiple comparisons test.


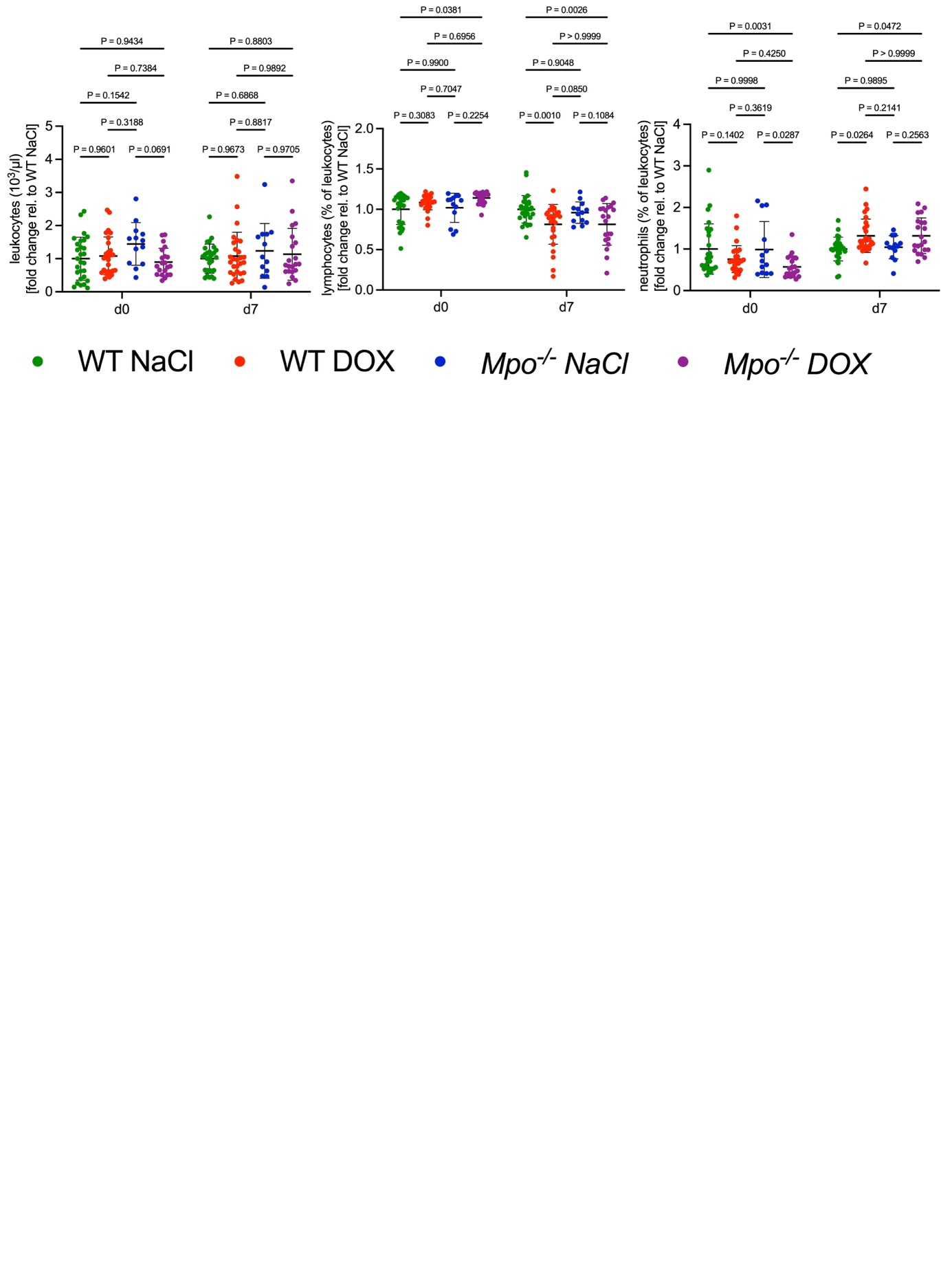


**Fig. S3** Normalized blood leukocytes (10^3^/µl), lymphocyte (% of leukocytes) and neutrophil frequencies (% of leukocytes). For this additional analysis, which was motivated by the observed batch effects between measurements at d0 (blood draw via facial vein puncture) and d7 (blood draw via cardiac puncture), values were normalized to the WT NaCl group at d0 and d7, respectively. d = day. n = 13-28 per group. Data are expressed as mean ± SD. Statistical significance was determined by two-way repeated measures ANOVA with Tukey’s multiple comparisons test.

**
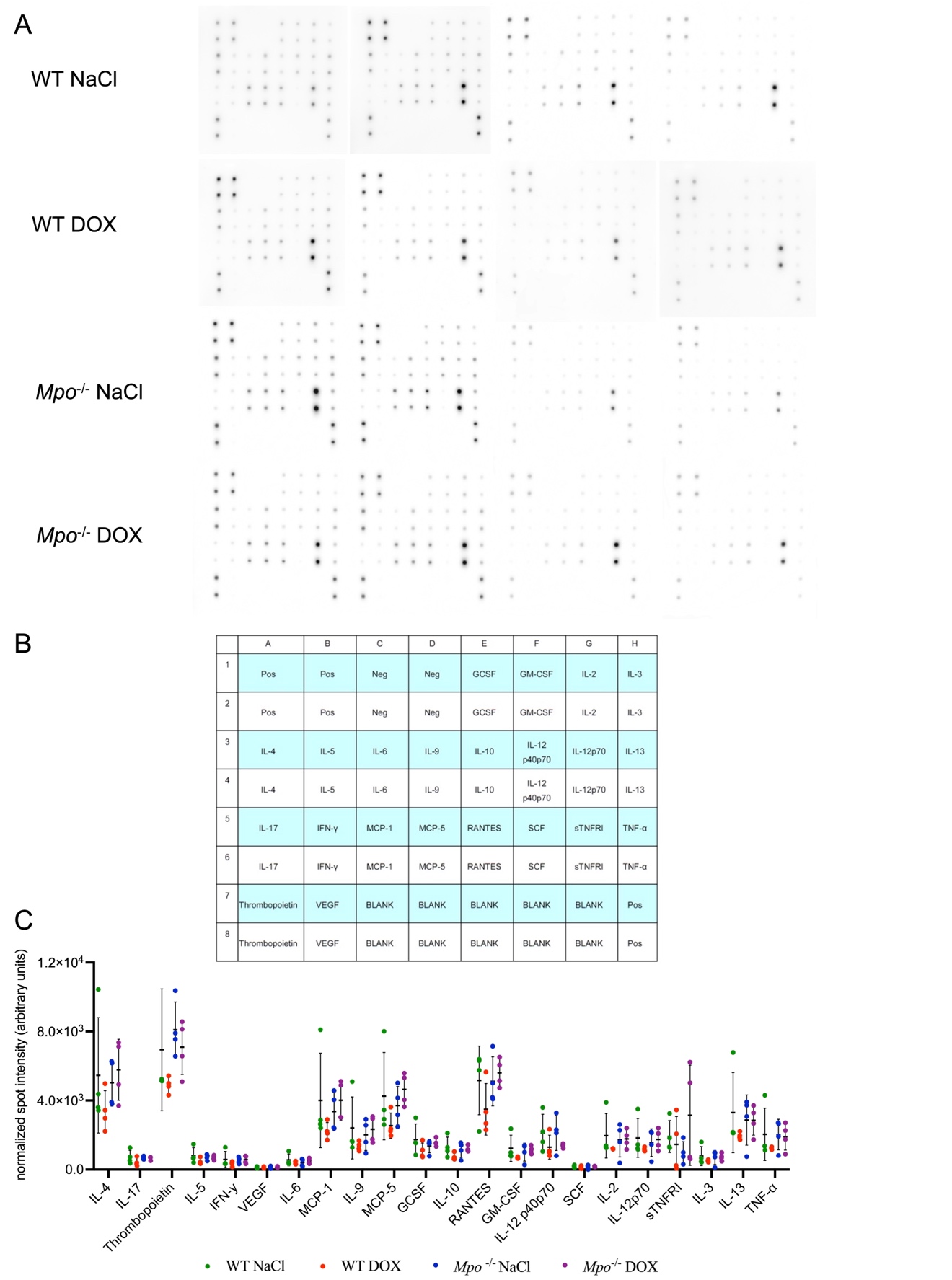
**

**Fig. S4** Plasma cytokine levels**.** (A) Images of cytokine antibody arrays. 4 membranes were stained per group and two samples were pooled on each membrane. (B) Array map. (C) Quantification of plasma cytokine levels (spot signal intensities, arbitrary units, normalized to internal positive control). (n = 4 replicates per group; two samples pooled per replicate). Data are expressed as mean ± SD. One-way ANOVA with Tukey’s multiple comparisons test did not yield any significant differences between the groups.


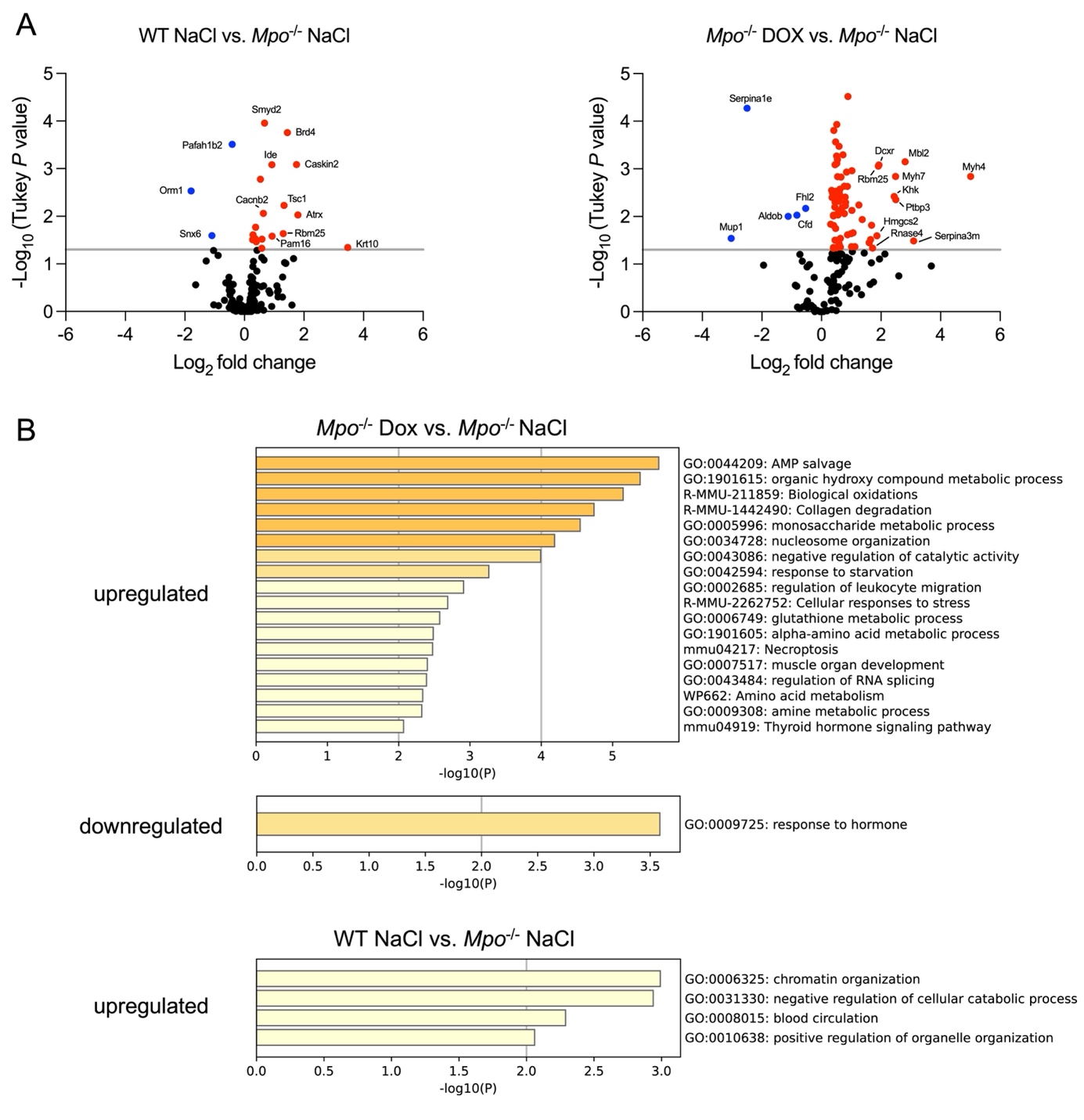


**Fig. S5** Cardiac protein expression. (A) Volcano plots of differentially expressed proteins between DOX- vs. NaCl-treated *Mpo^-/-^* mice and NaCl-treated WT vs. *Mpo^-/-^* mice. (D) Metascape pathway enrichment analysis (*Mpo^-/-^* DOX vs. *Mpo^-/-^* NaCl and WT NaCl vs. *Mpo^-/-^* NaCl). Downregulated pathways in WT NaCl vs. *Mpo^-/-^* NaCl were not detectable. n = 6 per group. Statistical significance of DE proteins was determined by one-way ANOVA (FDR-adjusted) with Tukey’s multiple comparison test.

**Fig. S6:** DOX does not impact cardiac expression of NADPH oxidases 1/3, xanthine oxidase and nitric oxide Synthases. (A) Cardiac mRNA expression of NADPH oxidase 1/3 (*Nox1/3*) and xanthine oxidase (*Xo*). (B) Xanthine oxidase enzyme activity as assessed by colorimetric oxidation assay. (C) Cardiac mRNA expression of nitric oxide synthase 1/2/3 (*Nos1//2/3*). Data are expressed as mean ± SD. n = 5-6 per group. Statistical significance was determined by one-way ANOVA with Tukey’s multiple comparisons.

**
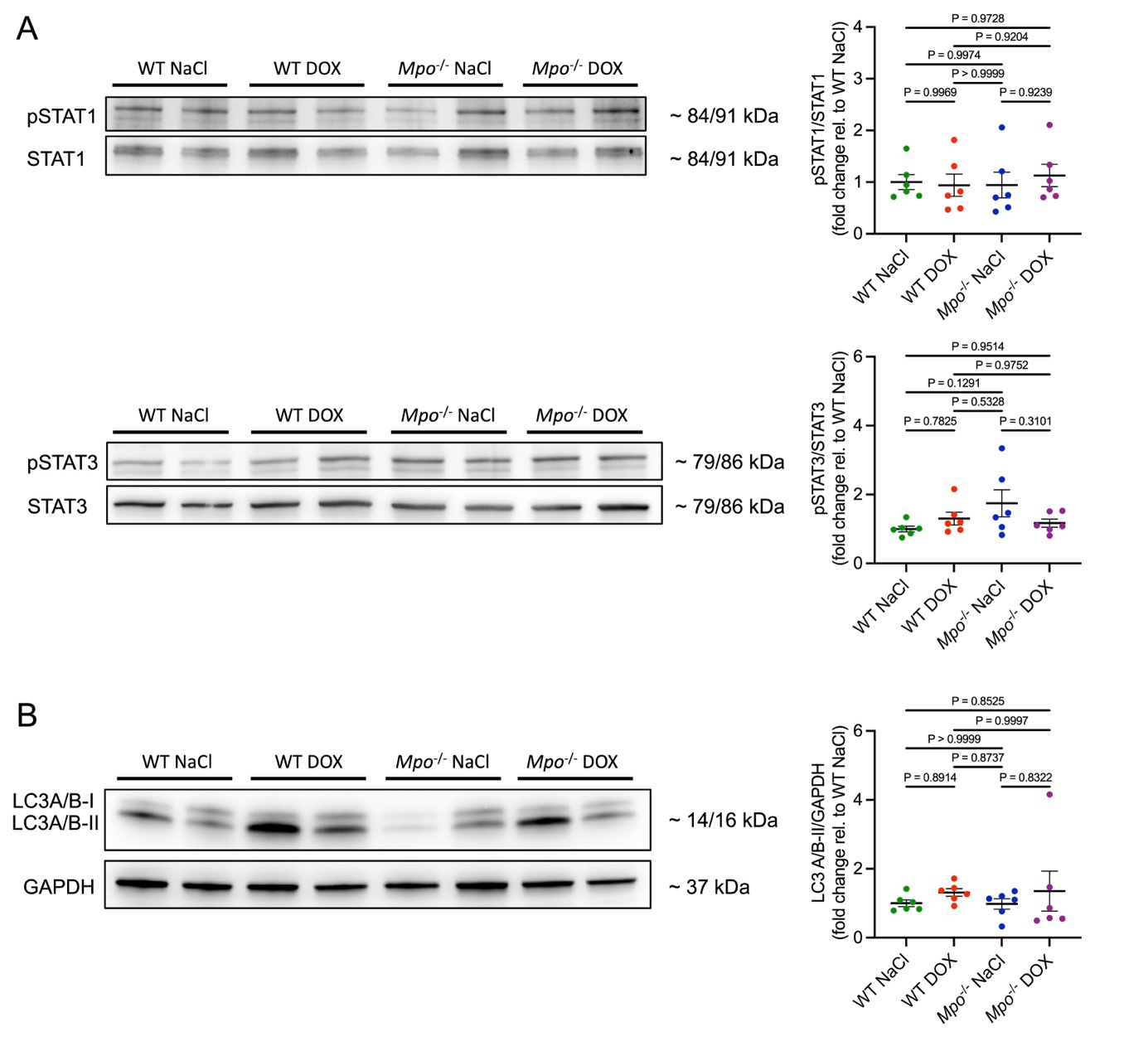
Fig. S7:** DOX neither influences cardiac STAT1/3 phosphorylation nor expression of autophagosome proteins LC3A/B. Representative immunoblots of phospho(p)/total STAT1, phospho(p)/total STAT3 (A) and LC3A/B (B) in cardiac tissue samples and corresponding quantification of protein expression. Data are expressed as mean ± SD. n = 6 per group. Statistical significance was determined by one-way ANOVA with Tukey’s multiple comparisons test.

**
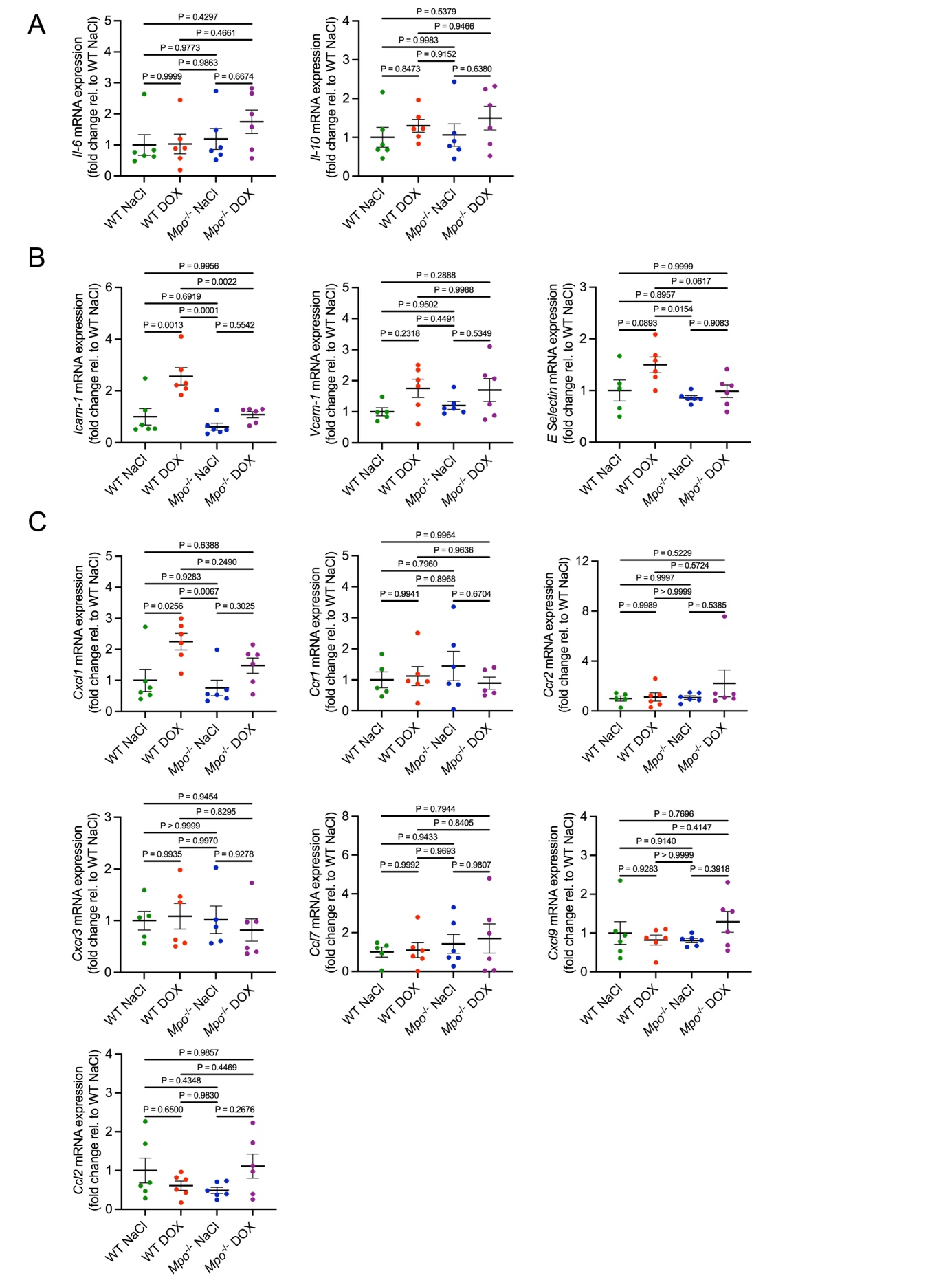
Fig. S8** DOX induces cardiac expression of endothelial adhesion molecules. Cardiac mRNA expression of several interleukins, endothelial adhesion molecules, chemokines and chemokine receptors. (A) Interleukins: Interleukin 6 (*Il-6*) and Interleukin 10 (*Il-10*). (B) Endothelial adhesion molecules: intercellular adhesion molecule 1 (*Icam-1*), vascular cell adhesion molecule 1 (*Vcam-1*), and E Selectin. (C) Chemokines and chemokine receptors: C-X-C motif chemokine ligand 1 (*Cxcl1*), C-C motif chemokine receptor type 1 (*Ccr1*), C-C motif chemokine receptor type 2 (*Ccr2*), C-X-C motif chemokine receptor 3 (*Cxcr3*), C-C motif chemokine ligand 7 (*Ccl7*), C-X-C motif chemokine ligand 9 (*Cxcl9*), C-C motif chemokine ligand 2 (*Ccl2*). Data are expressed as mean ± SD. n = 5-6 per group. Statistical significance was determined by one-way ANOVA with Tukey’s multiple comparisons test.

**
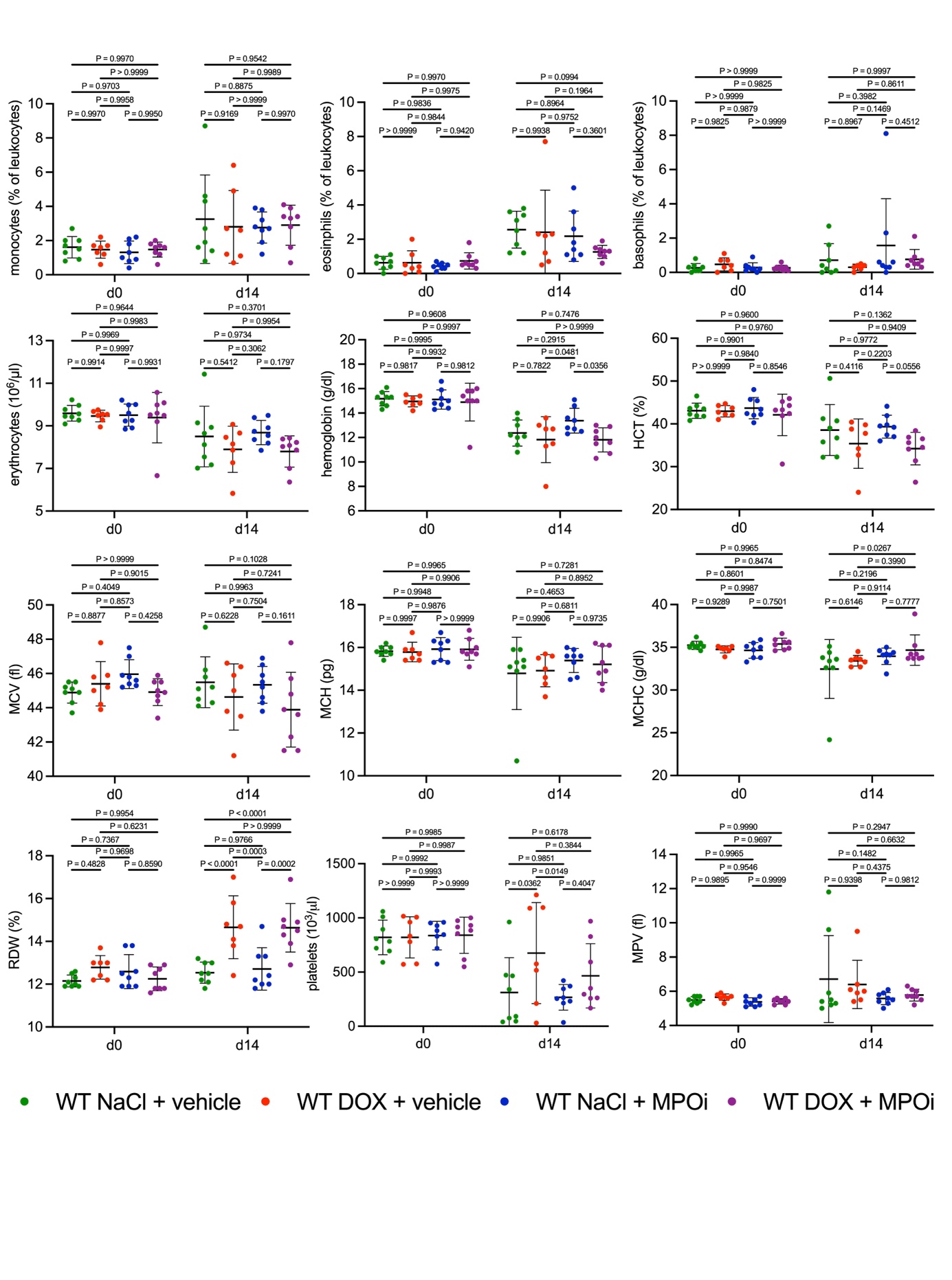
Fig. S9** Haematological parameters. Blood samples were taken before and 14 days after treatment (d = day). HCT = haematocrit, MCV = mean corpuscular volume, MCH = mean corpuscular haemoglobin, MCHC = mean corpuscular haemoglobin concentration, RDW = red cell distribution width. MPOi = MPO inhibitor 4-Aminobenzoic acid hydrazide. Vehicle = 10% DMSO dissolved in NaCl. Data are expressed as mean ± SD. n = 7-8 per group. Statistical significance was determined by two-way repeated measures ANOVA with Tukey’s multiple comparisons test.

**
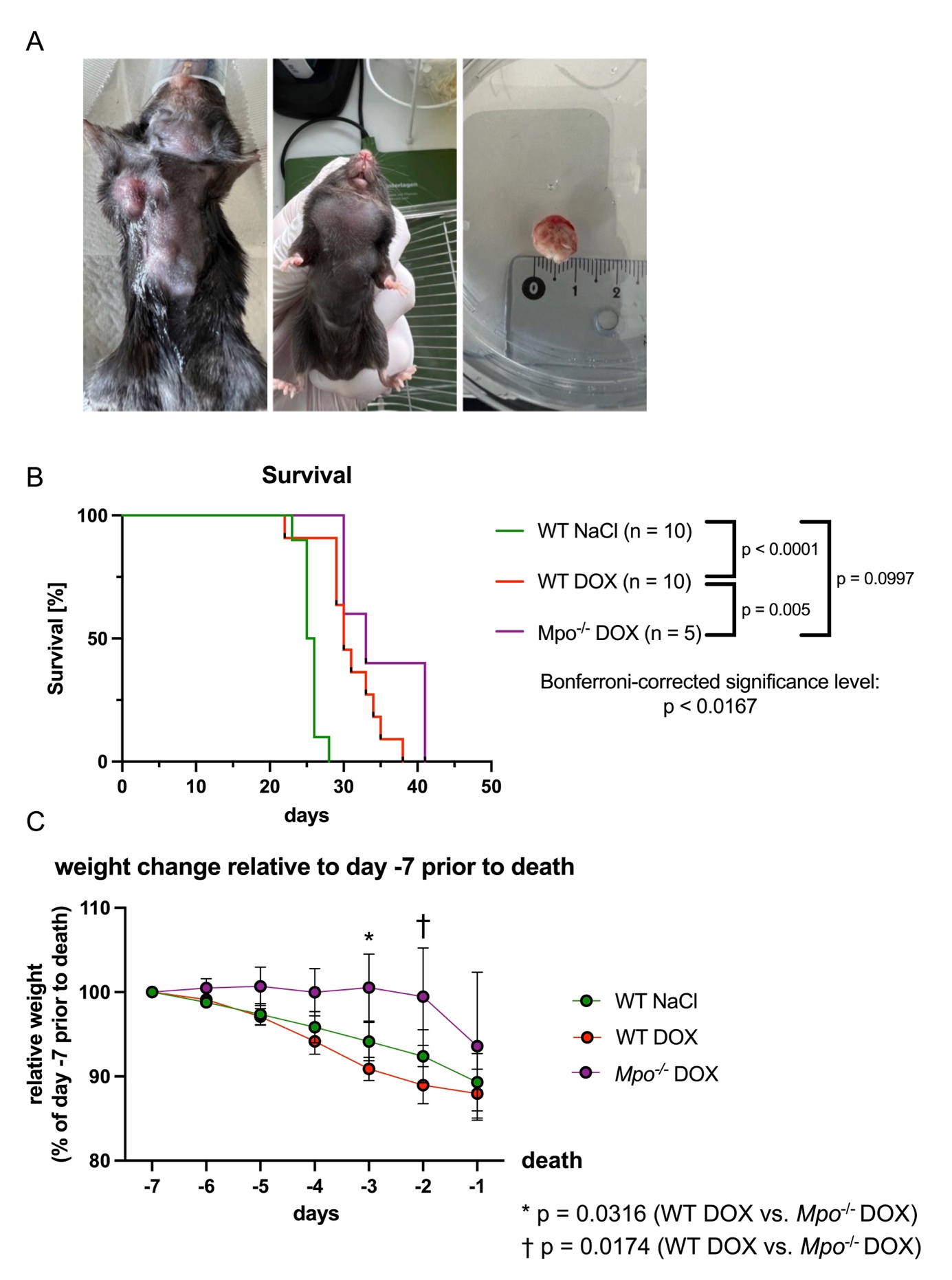
**

**Fig. S10** Tumour model to determine the impact of MPO deficiency on the anticancer efficacy of DOX. (A) Representative photograph showing tumours that developed after i.v. injection of 10 Mio. *Eμ-myc* B-cell lymphoma cells in WT animals. (B) Survival of mice after tumour cell implantation (n = 5-10 per group). A single bolus of DOX or NaCl was administered at day 7 after implantation. Statistical significance was determined by Log-rank (Mantel-Cox) test with post-hoc Bonferroni correction (adjusted significance level of P < 0.0167). (C) Weight changes relative to the weight seven days prior to death (n = 5-10 per group). Data are expressed as mean ± SEM. Statistical significance was determined by two-way repeated measures ANOVA with Tukey’s multiple comparisons test.

**
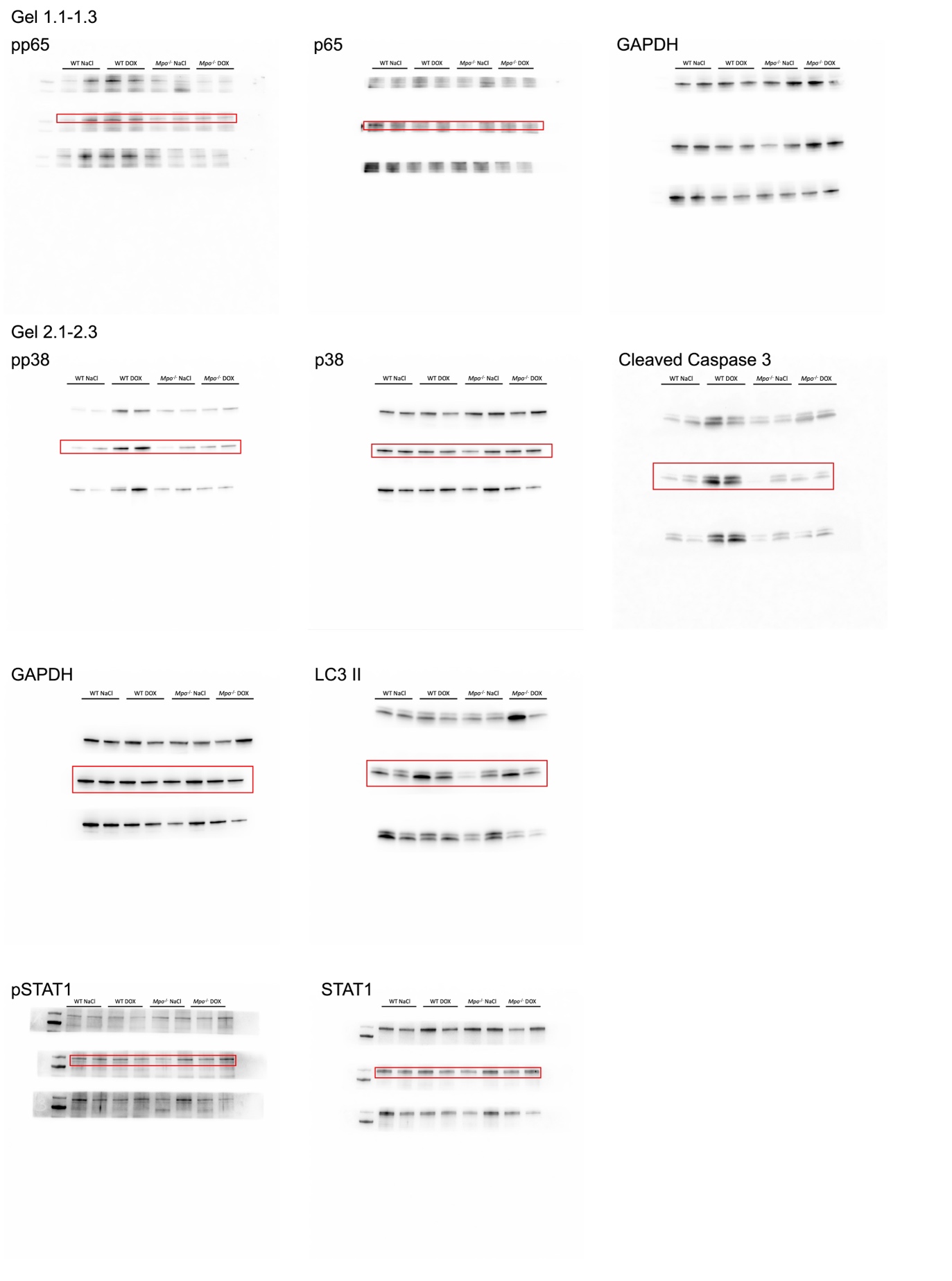
Fig. S11** Uncropped immunoblot gels (gels 1 and 2). In all cases, 24 samples (n = 6 per group) were loaded on three gels and membranes were imaged together (8 samples per gel; for image acquisition, the first, second, and third membrane were placed at the top, in the middle, and at the bottom, respectively). Representative lanes shown in the manuscript are indicated by red rectangles.

**
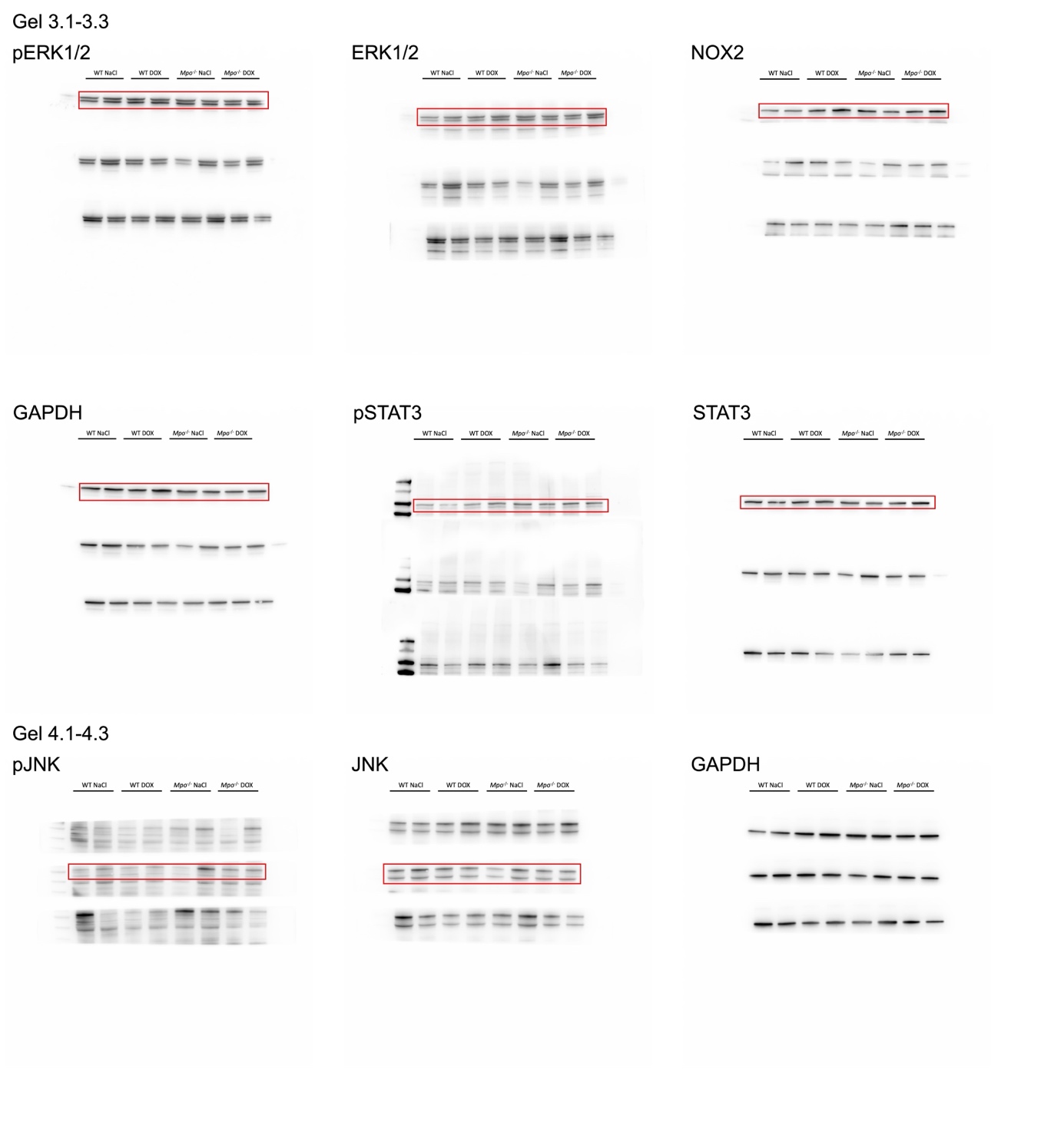
Fig. S12** Uncropped immunoblot gels (gels 3 and 4). In all cases, 24 samples (n = 6 per group) were loaded on three gels and membranes were imaged together (8 samples per gel; for image acquisition, the first, second, and third membrane were placed at the top, in the middle, and at the bottom, respectively). Representative lanes shown in the manuscript are indicated by red rectangles.

**
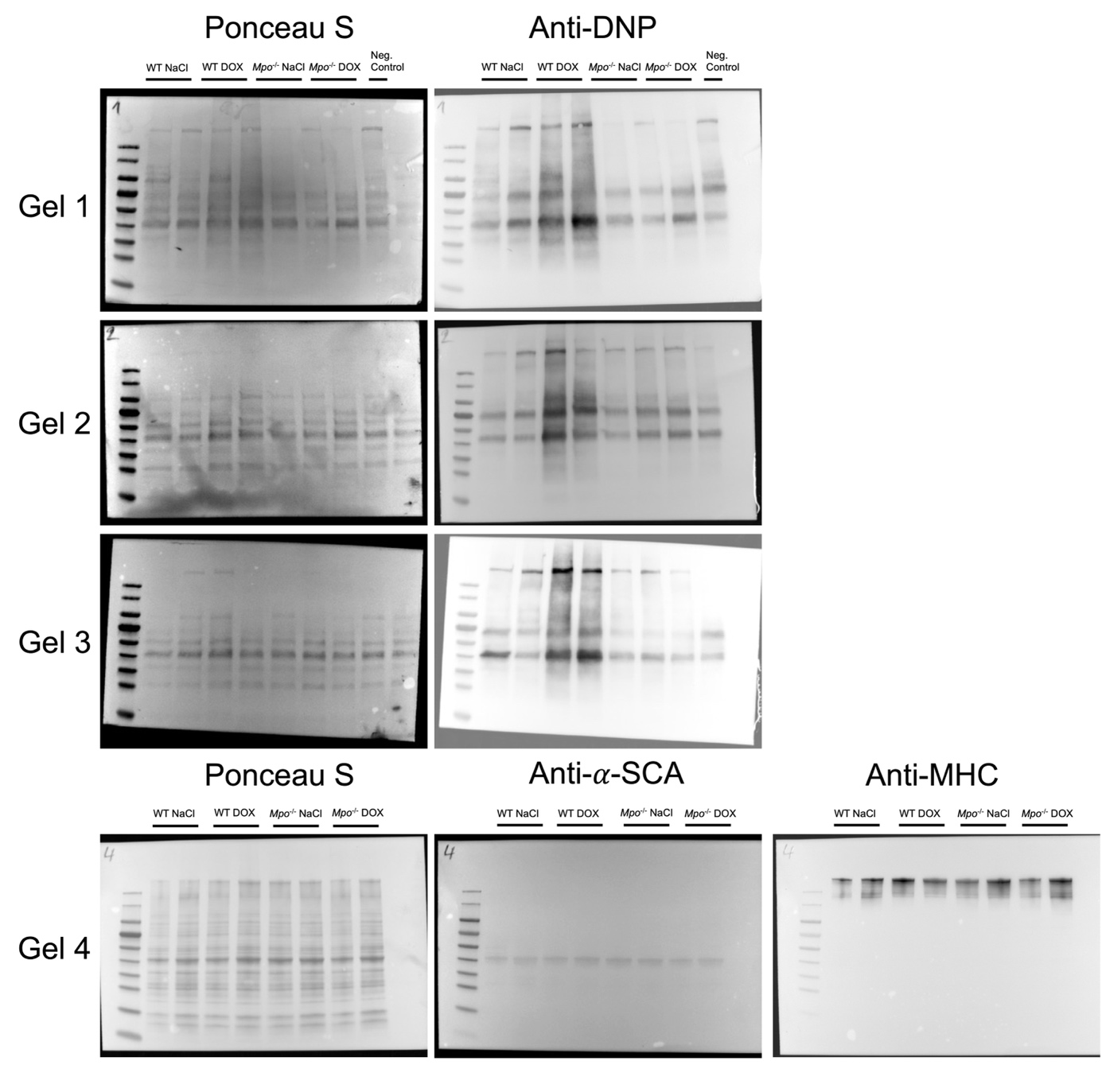
Fig. S13** Uncropped protein carbonyl immunoblot gels. 24 samples (n = 6 per group) and were loaded on three gels (8 samples per gel). Additionally, one unstained control was included on each gel. Ponceau S staining of the gels was performed as loading control. Samples from Gel 3 were loaded on another gel (Gel 4) and stained with anti-𝛼-sarcomeric Actin (𝛼-SCA) and anti-myosin heavy chain (MHC) antibodies to verify that two of the major bands detected in the anti-DNP staining corresponded to 𝛼-SCA and MHC, respectively. Gels 3 and 4 are shown as representative images in the manuscript.

1. **Supplementary Tables**

| **Target Protein** | **Species** | **Manufacturer** | **Catalogue-No.** | **Dilution for Immunoblot** | **Dilution for**  **IF-staining** |
| --- | --- | --- | --- | --- | --- |
| α-sarcomeric Actin | mouse | Sigma-Aldrich | SAB4200689 | 1/1000 | - |
| Cleaved Caspase 3 | rabbit | Cell Signaling | 9665 | 1/1000 | - |
| ERK1/2 | rabbit | Cell Signaling | 4695 | 1/1000 | - |
| pERK1/2 | rabbit | Cell Signaling | 9101 | 1/1000 | - |
| GAPDH | rabbit | Cell Signaling | 2118 | 1/7500 | - |
| JNK | rabbit | Cell Signaling | 9252 | 1/1000 | - |
| p-JNK | mouse | Cell Signaling | 9255 | 1/1000 | - |
| LC3A/B | rabbit | Cell Signaling | 4108 | 1/1000 | - |
| MHC | mouse | Abcam | ab50967 | 1/1000 | - |
| NOX2 | rabbit | Abcam | ab129068 | 1/5000 | - |
| p38 | rabbit | Cell Signaling | 9212 | 1/1000 | - |
| pp38 | rabbit | Cell Signaling | 9211 | 1/1000 | - |
| p65 | rabbit | Cell Signaling | 7970 | 1/1000 | - |
| pp65 | rabbit | Cell Signaling | 3033 | 1/1000 | - |
| STAT1 | rabbit | Cell Signaling | 9172 | 1/1000 | - |
| pSTAT1 | rabbit | Cell Signaling | 9171 | 1/1000 | - |
| STAT3 | rabbit | Cell Signaling | 30835 | 1/1000 | - |
| pSTAT3 | rabbit | Cell Signaling | 9145 | 1/1000 | - |
| 3-nitrotyrosine | goat | LS BioSience | LS-C124272 | - | 1/300 |
| CD68 | rat | BioLegend | 137020 | - | 1/200 |
| F4/80 | rat | Abcam | ab6640 | - | 1/200 |
| Ly6G | rat | BioLegend | 127636 | - | 1/200 |

**Table S1.** Antibody list.

| **Target** | **host** | **forward Primer** | **reverse Primer** |
| --- | --- | --- | --- |
| *Anp/Anf* | mouse | TCGTCTTGGCCTTTTGGCT | TCCAGGTGGTCTAGCAGGTTCT |
| *Bnp* | mouse | ATGGATCTCCTGAAGGTGCTG | GTGCTGCCTTGAGACCGAA |
| *Ccl-2* | mouse | CAGCACCAGCCAACTCTCAC | ATTCCTTCTTGGGGTCAGCA |
| *Ccl-7* | mouse | GAGGATCTCTGCCACGCTTC | AGGCTTTGGAGTTGGGGTTT |
| *Ccr-1* | mouse | GTTGGGACCTTGAACCTTGA | CCCAAAGGCTCTTACAGCAG |
| *Ccr-2* | mouse | CCACACCCTGTTTCGCTGTA | TGCATGGCCTGGTCTAAGTG |
| *Col1a1* | mouse | CGACCTCAAGATGTGCCACT | ACTCGAACGGGAATCCATCG |
| *Col3a1* | mouse | GCCTTCTACACCTGCTCCTG | TTCCTCCCACTCCAGACTTG |
| *Ctgf* | mouse | GAAGGGCAAAAAGTGCATCC | TGTAATGGCAGGCACAGGTC |
| *Cxcl-9* | mouse | CTGGAGCAGTGTGGAGTTCG | GGCAGGTTTGATCTCCGTTC |
| *Cxcr-3* | mouse | GGCGTTTTCGAGCTATGAGG | ATGCCCGAGGTGACTGACTT |
| *E-Selectin* | mouse | GGACACCACAAATCCCAGTCTG | TCGCAGGAGAACTCACAACTGG |
| *Icam-1* | mouse | GACTGCTTGGGGAACTGGAC | GCCCACAATGACCAGCAGTA |
| *Il-10* | mouse | AACTGCACCCACTTCCCAGT | GCCTGGGGCATCACTTCTAC |
| *Il-18* | mouse | GACAGCCTGTGTTCGAGGATATG | TGTTCTTACAGGAGAGGGTAGAC |
| *Il-1β* | mouse | GACCTTCCAGGATGAGGACA | AGGCCACAGGTATTTTGTCG |
| *Il-6* | mouse | TACCACTTCACAAGTCGGAGGC | CTGCAAGTGCATCATCGTTGTTC |
| *Cxcl1* | mouse | CACACTCCAACACAGCACCA | AGCTTCAGGGTCAAGGCAAG |
| *Nlrp3* | mouse | CTGGAGCAAAGGCTTGTGTG | CCAGAGGCCCAGAGAGAGAA |
| *TGF-β receptor 1* | mouse | TGATACGCCTGAGTGGCTGTCT | CACAAGAGCAGTGAGCGCTGAA |
| *Tnf-α* | mouse | AAGTGGAGGAGCAGCTGGAG | CGGCAGAGAGGAGGTTGACT |
| *Vcam-1* | mouse | AGGGTGGTGCTGTGACAATG | ACTTGTGCAGCCACCTGAGA |
| *Xanthinoxidase* | mouse | ACAAAGGACCAGACGATTGC | CCTCTAGATGCGAGGGTCAG |
| *Nox1* | mouse | CAGGGGACTGGACACGAAAT | AAAGGCAGGTTCCCAGGATT |
| *Nox2* | mouse | GAGGCAGAACCAACACTTAACC | TGAAGAGATGTGCAATTGTGTG |
| *Nox3* | mouse | GCCGTGTGCCTGAATTTTA | ATGATGAAGACCAGAGCCAGA |
| *Nos1* | mouse | ACCAGCACCTTTGGCAATGGAG | GAGACGCTGTTGAATCGGACCT |
| *Nos2* | mouse | GAGACAGGGAAGTCTGAAGCAC | CCAGCAGTAGTTGCTCCTCTTC |
| *Nos3* | mouse | GAGATCAAAGGGCTACAACCTG | TAGAGATGGTCCAGTTGGGAG |
| *β -Actin* | mouse | GCTGTATTCCCCTCCATCG | GGGGTGTTGAAGGTCTCAAA |

**Table S2.** Primer list
